# Significance of Immune-Related Genes in the Diagnosis and Classification of Intervertebral Disc Degeneration

**DOI:** 10.1155/2022/2616260

**Published:** 2022-08-30

**Authors:** Bo Wu, Xinzhou Huang, Mu Zhang, Wei Chu

**Affiliations:** ^1^Department of Orthopedics, The First People's Hospital of Jingzhou (First Affiliated Hospital of Yangtze University), Jingzhou, Hubei, China; ^2^Department of Anesthesia, The First People's Hospital of Jingzhou (First Affiliated Hospital of Yangtze University), Jingzhou, Hubei, China

## Abstract

**Background:**

With the extensive development of intervertebral disc degeneration (IDD) research, IDD has been found to be a complex disease associated with immune-related gene (IRGs) changes. Nonetheless, the roles of IRGs in IDD are unclear.

**Methods:**

In our study, 11 IRGs were chosen using differential analysis between nondisc degeneration and degenerative patients from the GEO database. Then, we utilized a random forest (RF) model to screen six candidate IRGs to predict the risk of IDD. A nomogram was developed on the basis of six candidate IRGs, and DCA showed that patients could benefit from the nomogram. Based on the selected significant IRGs, a consensus clustering approach was used to differentiate disc degeneration patients into two immune patterns (immune cluster A and B). The PCA algorithm was constructed to compute immune scores for every sample, to quantify immune patterns. The immune scores of immune cluster B patients were higher than those of immune cluster A.

**Results:**

Through differential expression analysis between healthy and IDD samples, 11 significant IRGs (CTSS, S100Z, STAT3, KLRK1, FPR1, C5AR2, RLN1, IFGR2, IL2RB, IL17RA, and IL6R) were recognized through significant IRGs. The “Reverse Cumulative Distribution of Residual” and “Boxplots of Residual” indicate that the RF model has minimal residuals. The majority of samples in the model have relatively small residuals, demonstrating that the model is better. Besides, the nomogram model was constructed based on importance and the IRGs with importance scores greater than 2 (FPR1, RLN1, S100Z, IFNGR2, KLRK1, and CTSS). The nomogram model revealed that decision-making based on an established model might be beneficial for IDD patients, and the predictive power of the nomogram model was significant. In addition, we identified two different immune cluster patterns (immune cluster A and immune cluster B) based on the 11 IRGs. We found that immune cluster A had significantly higher levels of MDSC, neutrophil, plasmacytoid dendritic cell, and type 17 T helper cell expression than immune cluster B. And we calculated the score for each sample to quantify the gene patterns. The patients in immune cluster B or gene cluster B had higher immune scores than those in immune cluster A or gene cluster A.

**Conclusion:**

In conclusion, IRGs play an extremely significant role in the occurrence of IDD. Our study of immune patterns may guide the strategies of prevention and treatment for IDD in the future.

## 1. Introduction

Intervertebral disc degeneration (IDD) is believed to be the major cause of low back pain, placing a heavy load on healthcare systems worldwide [1]. The mechanisms of IDD pathogenesis are considerably complex and many factors can contribute to IDD, including genetics, age, and poor lifestyle habits [2]. Until now, good nonsurgical treatment strategies to reverse IDD have not been available, mainly owing to the poorly understood mechanism of IDD and a lack of effective targets [3]. Treatment choices for low back pain arising from IDD are extremely restricted owing to the complex and ill-defined pathology of the disease [4]. Conventional medical management of low back pain consists of nonpharmacological (e.g., physical therapy) and pharmacological treatments. Surgery may be taken into consideration when conventional treatment does not relieve the pain for more than three months [5]. Nevertheless, surgical operations like spinal fusion are intrusive and usually require a long-term postoperative recovery time with a significant risk of surgical complexity and a high recurrence rate after surgery [6]. Early diagnosis and prompt treatment of IDD can significantly slow down the progression of IDD and reduce the incidence of disability. Hence, selecting diagnostic genes related to IDD, probing subtype classification, and illuminating the potential pathogenesis of IDD can prevent and treat IDD significantly and may offer novel avenues for clinical treatment of IDD.

In the last few years, much research has confirmed that immune-related genes (IRGs) have a significant role in the occurrence of IDD. With the extensive development of IDD research, IDD is now a complex disease associated with IRG changes. For example, in general, in the late stage of IDD, it is often present as an annulus fibrosus tear, nucleus pulposus herniation, and sciatica [7]. When lumbar disc herniation occurs, mechanical compression can lead to low back pain. In addition to physical compression, research that has been shown suggested that the autoimmune response of the nucleus pulposus (NP) is a critical intermediate in the neurogenic pain of lumbar disc herniation, and research has shown that diverse kinds of activated immune cells and inflammatory factors accumulate in the NP nerve root region [8]. Immune cells and inflammatory factors constitute a complex area that contributes to immune stress in nerve roots [9–12]. In addition, angiogenesis and neurogenesis through blood channel infiltration and neural sensitization can also exacerbate this condition [13]. Therefore, IRGs are of great significance as biomarkers for IDD. Therefore, it is crucial to identify IDD at an early stage, and early screening and effective prevention of high-risk groups from the perspective of IRGs will have far-reaching effects on the management of IDD.

In the research, we used bioinformatics methods to explore the function of IRGs in the diagnosis and classification of IDD from the Gene Expression Omnibus (GEO) database. First, we identified differential expression of IRGs from the GSE124272 and GSE150408 datasets. Then, we screened six candidate IRGs associated with IDD using a random forest (RF) model and developed a nomogram for predicting the incidence of IDD. In addition, we divided gene expression profiles into two immune clusters and explored their relationship with infiltrating immune cells. Finally, we further characterized the association between the two clusters and cytokines. We found that the immune patterns could distinguish IDD patients from normal people and provide new directions in the prevention and treatment of IDD.

## 2. Materials and Methods

### 2.1. Data Collection

Blood samples from 25 healthy controls and 25 IDD patients were collected by us from GSE124272 and GSE150408 in the GEO database (https://www.ncbi.nlm.nih.gov/geo/) [14, 15]. The two datasets were merged, normalized, and batch corrected; and differential expression analysis was performed by using the “limma” package. We acquired 2483 IRGs from the ImmPort database (http://www.immport.org) and identified 11 differentially expressed IRGs (*p* < 0.001) (Supplementary Table 1 and 2) [16]. Person correlation analysis was used for the correlation analysis.

### 2.2. Establishment of Models

We established RF and support vector machine (SVM) models to predict the training model for the occurrence of IDD. “Inverse Cumulative Distribution of Residuals,” “Box Plot of Residuals,” and receiver operating characteristic (ROC) curves were constructed to assess the models. RF is a compositionally supervised learning method, which is regarded as an expansion of decision trees. We utilized the “Random Forest” package to develop the RF model and chose candidate IRGs to predict the occurrence of IDD. In addition, we set the *n*-trees and *m*-trees at 500 and 3, respectively. Then, we analyzed the significance of IRGs, and appropriate IRGs were selected by 10-fold cross-validation. The precision of the model in choosing various numbers of IRGs is presented by the *y*-axis. On the basis of the structural risk minimization principle in statistical learning theory, SVM has been considered as a supervised machine learning algorithm. Each data point as a point in *n*-dimensional space was plotted. And we were able to identify the most suitable hyperplane to distinguish the two classes (normal and degenerated discs) well [17–19]. Finally, we utilized ROC curves and area under the curve (AUC) to evaluate the predictive accuracy of these 11 IRGs [20].

### 2.3. Construction of Nomogram

We developed a nomogram on the basis of candidate IRGs to predict the incidence of IDD. The concordance of our predicted values with the realistic relationship was evaluated by the calibration curve. We performed the decision curve analysis (DCA) and plotted a clinical impact curve to evaluate if the decision on the basis of the model was beneficial to the IDD patients [21].

### 2.4. Identification of Molecular Subtypes

Consensus clustering, as a resampling-based method, is often applied to recognize each member and their subgroup number and validate the rationality of the clustering algorithm. The distinct immune patterns based on the significant IRGs were identified by performing the consensus clustering method by using the “Consensus Cluster Plus” package.

### 2.5. Performance of Gene Ontology Functional Enrichment Analysis

The 220 different expressed genes (DEGs) between distinct immune patterns were screened by the “limma” package (*p* < 0.05 and log FC > 0.585). We used gene ontology (GO) enrichment analysis to investigate the potential mechanism of the DEGs via using the “cluster Profiler” package and plotted an enrichment circle diagram to make results visualized [22–24].

### 2.6. Estimation of the Immune Gene Signature

We utilized principal component analysis (PCA) to compute immune scores for each sample to quantify different patterns. The principal component 1 (PC1) and PC2 were chosen as the signature scores. And immune scores for each IDD patient were calculated using the following formula: Immune Score = *Σ*(PC1_*i*_ + PC2_*i*_), where *i* is the expression of IRGs [25].

### 2.7. Estimation of Immune Cell Infiltration

The single-sample gene-set enrichment analysis (ssGSEA) was employed to measure the relative abundance of immune cell samples [26]. And the gene set for marking each immune cell type was obtained from the study of Charoentong et al. [27].

## 3. Results

### 3.1. Landscape of the IRGs in IDD

**Figure 1:**
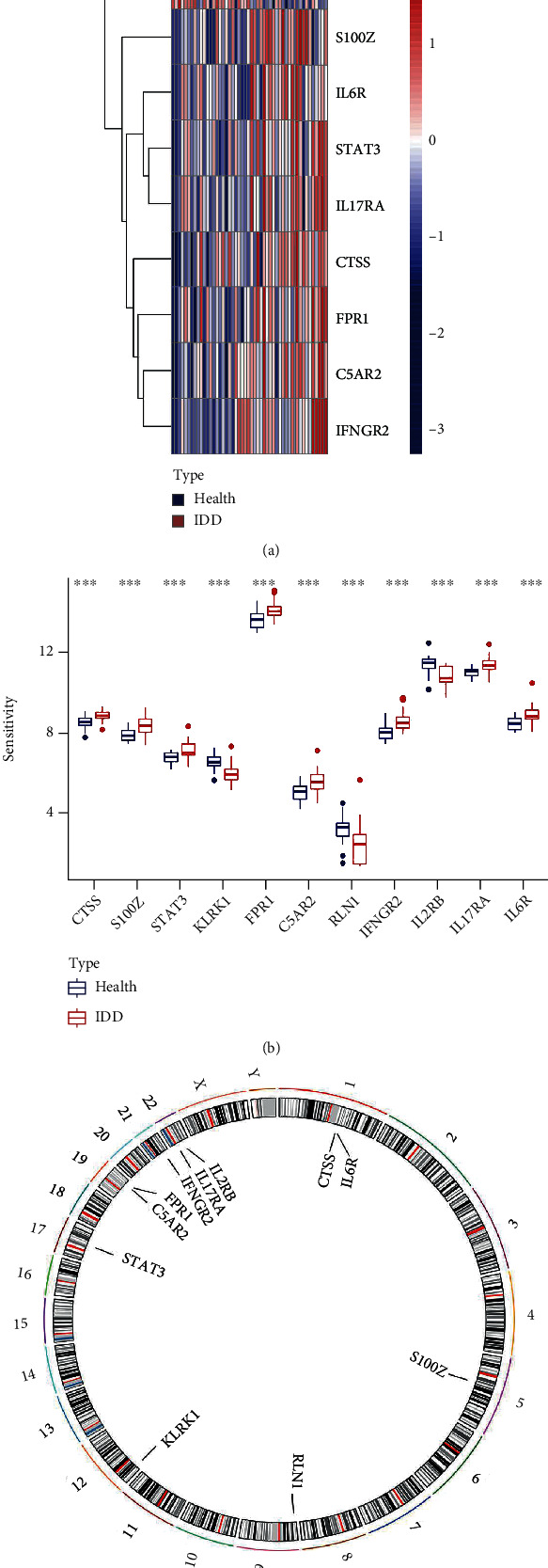
(a) Expression heat map of the 11 IRGs in non-disc degeneration and degeneration patients. (b) Differential expression histogram of the 11 IRGs identified between nondisc degeneration and IDD patients. (c) Chromosomal positions of the 11 IRGs.

11 significant IRGs (CTSS, S100Z, STAT3, KLRK1, FPR1, C5AR2, RLN1, IFGR2, IL2RB, IL17RA, and IL6R) were filtered and visualized (*p* < 0.001). KLRK1, RLN1, and IL2RB were decreased in IDD patients, while the other significant IRGs were overexpressed in IDD patients compared to nondisc degeneration patients (Figures 1(a) and 1(b)). The chromosomal positions of 11 IRGs were visualized using the “RCircos” package (Figure 1(c)).

### 3.2. Correlation between IRGs in IDD

**Figure 2:**
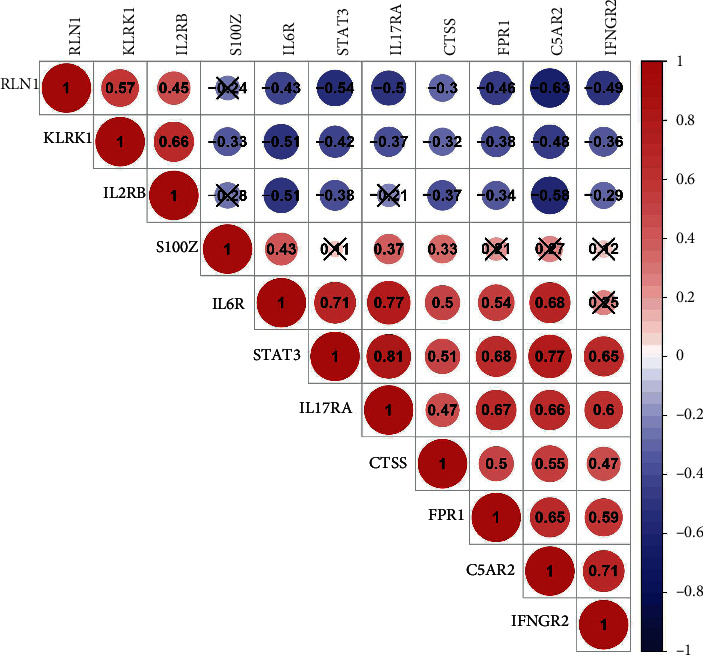
We obtained reciprocal correlations between the 11 candidate IRGs by R package corrplot, with numbers for correlation coefficients, red for positive association, and blue for negative. ×: *p* > 0.05.

To investigate the association between 11 IRGs in IDD, we performed R package corrplot for correlation analysis (Figure 2). The expression levels of STAT3 in IDD patients had a highly positive association with IL17RA with a correlation coefficient of 0.81, IL6R with IL17RA, and STAT3 with C5AR2 (both 0.77). And a negative relationship is between C5AR2 and RLN1, with a correlation coefficient of -0.63, and IL2RB with C5AR2, RLN1 with STAT3 (0.58 and 0.57, respectively) in IDD patients.

### 3.3. Construction of the RF and SVM Models

**Figure 3:**
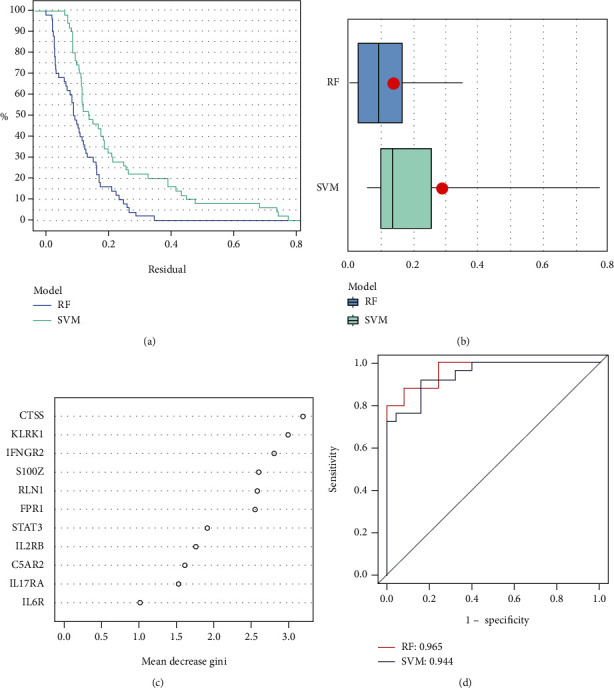
(a) Reverse cumulative distribution of residual. (b) Boxplots of residual. (c) 11 IRGs were visualized after ranking genes based on importance, and six IRGs (including FPR1, RLN1, S100Z, IFNGR2, KLRK1, and CTSS) with importance scores greater than 2. (d) The AUC value of the ROC curve indicated that the RF model (0.965) has higher accuracy than the SVM model (0.944).

We established the RF and SVM models to choose key IRGs to predict the incidence of IDD. Both “Reverse Cumulative Distribution of Residual” (Figure 3(a)) and “Boxplots of Residual” (Figure 3(b)) indicate that the RF model has minimal residuals. The majority of samples in the model have relatively small residuals, demonstrating that the model is better. Therefore, the RF model was believed to be the best model to predict the occurrence of IDD. We visualized the 11 IRGs after ranking genes based on importance, and the IRGs with importance scores greater than 2 (FPR1, RLN1, S100Z, IFNGR2, KLRK1, and CTSS) (Figure 3(c)). Finally, the ROC curve was plotted to evaluate the model, and the AUC value of the ROC curve also indicated that the RF model has higher accuracy than the SVM model (Figure 3(d)).

### 3.4. Construction of the Nomogram

**Figure 4:**
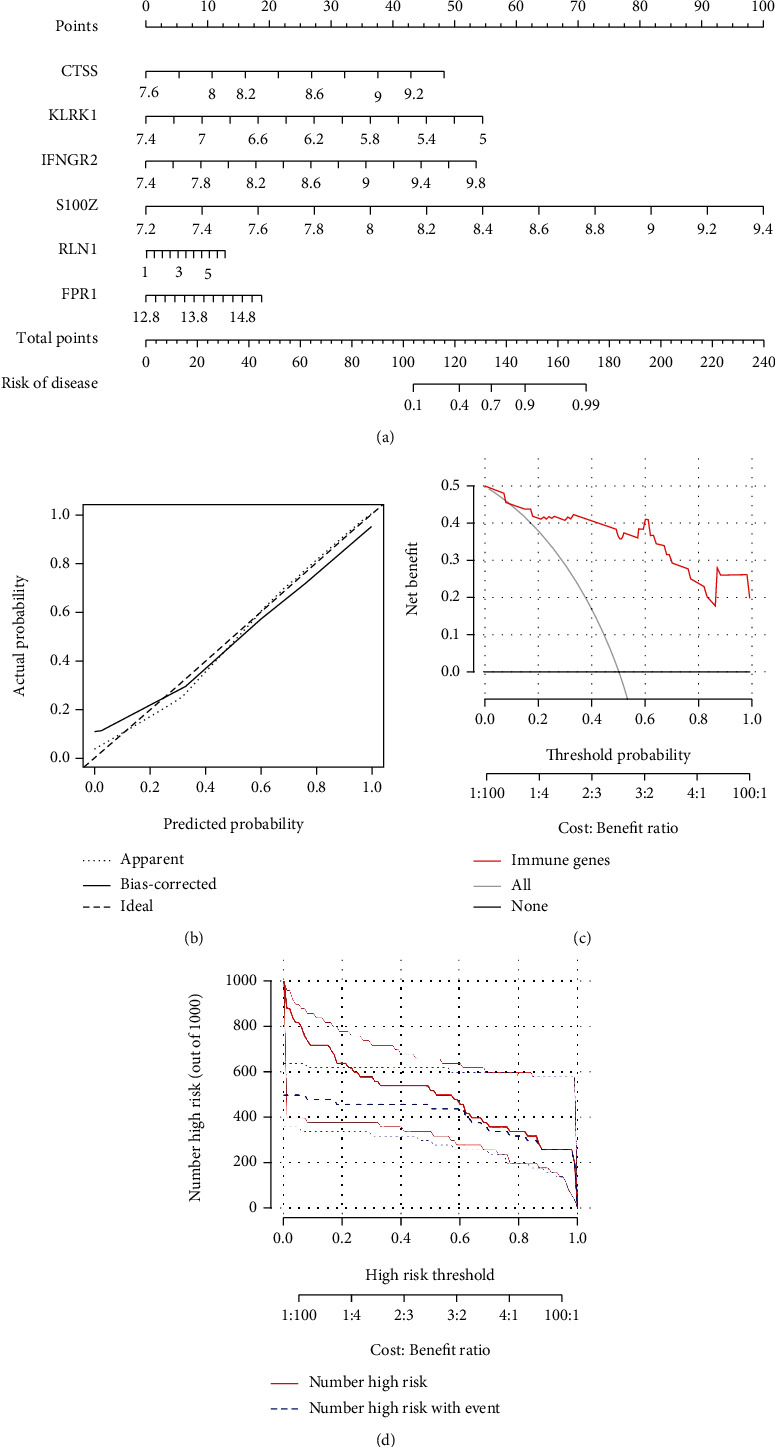
(a) A nomogram on the basis of six candidate IRGs was developed to predict the prevalence of IDD in patients. (b) We confirm that the nomogram predictions are accurate by the results of the calibration curve. (c) The DCA curve suggests that decision-making on the basis of the nomogram may benefit patients with IDD because the red lines are consistently maintained above the gray and black lines of 0 to 1. (d) The significant predictive ability of the nomogram was demonstrated by the clinical impact curves.

The “rms” package was used to create a nomogram on the basis of the six IRGs to forecast the incidence of IDD patients (Figure 4(a)). The accuracy of the nomogram's predictivity was demonstrated via calibration curves (Figure 4(b)). From 0 to 1, the red line in the DCA curve remained above the gray and black lines, showing that IDD patients may benefit from judgments on the basis of the nomogram (Figure 4(c)). The clinical impact curve demonstrated the amazing predictive potential of the nomogram (Figure 4(d)). Additionally, the AUC values for the six IRGs were over 0.75, showing good sensitivity and specificity of the signature for the prevalence of IDD (Supplementary Figure 1).

### 3.5. Two Distinct Pyroptosis Patterns

**Figure 5:**
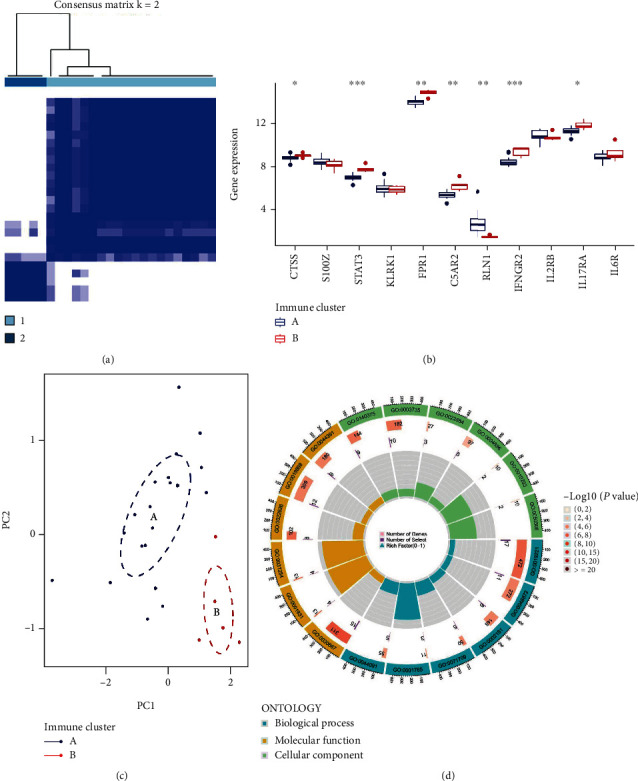
(a) Consensus clustering in the “ConsensusClusterPlus” package was used to identify different immune patterns (immune clusters A and B) on the basis of 11 IRGs. (b) Differential expression levels between the 11 significant IRGs between the two clusters are shown by histograms, and the results suggested that CTSS, STAT3, FPR1, C5AR2, IFGR2, and IL17RA displayed higher expression levels in immune cluster B than in immune cluster A, while RLN1 showed the opposite. S100Z, KLRK1, IL2RB, and IL6R demonstrated no significant differences between immune cluster A and B. (c) 11 significant IRGs can completely distinguished the two patterns by the PCA algorithm. (d) The GO functional enrichment analysis and the enrichment circle plot visualization results were used to understand the possible mechanism of the different immune patterns in IDD.

Using the “ConsensusClusterPlus” package, the consensus clustering approach was employed to distinguish between two immune patterns (immune cluster A and B) on the basis of the 11 IRGs (Figure 5(a) and Supplementary Figure 2). Twenty instances were found in immune cluster A, while five cases were found in immunological cluster B. The differential expression levels of the 11 IRGs between the two clusters were then shown by plotting the histogram. Immune cluster B revealed greater expression levels than immune cluster A for CTSS, STAT3, FPR1, C5AR2, IFGR2, and IL17RA, but RLN1 demonstrated the reverse (Figure 5(b)). Immune clusters A and B did not differ significantly according to S100Z, KLRK1, IL2RB, or IL6R.  Figure 5(c)'s PCA algorithm findings show that 11 IRGs can clearly discriminate between the two immune patterns.

**Figure 6:**
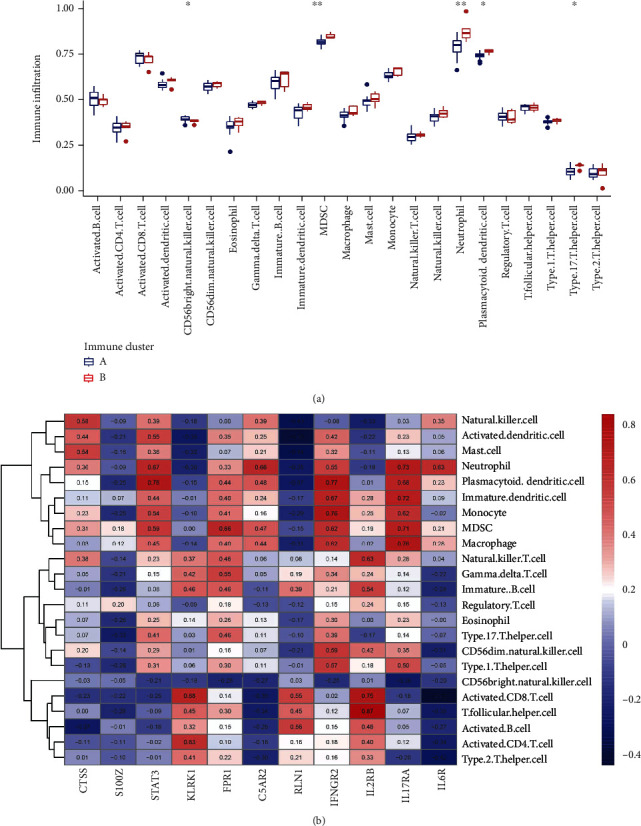
(a) Association between infiltrating immune cells and immune patterns. MDSC, neutrophil, plasmacytoid dendritic cell, and type 17 T helper cell were significantly highly expressed in immune cluster B, and CD56 bright natural killer cell was significantly highly expressed in immune cluster A. (b) The ssGSEA was used to compute the abundance of immune cells and to assess the association between 11 IRGs and immune cells.

An enrichment circle diagram was created using the findings of the GO functional enrichment analysis in order to better understand the potential mechanism of two immune patterns in IDD (Figure 5(d)). In the GO enrichment analysis of DEGs, biological processes (BP) terms were correlated with GO: 0019221 (cytokine-mediated signaling pathway) and GO: 0048872 (homeostasis of number of cells); cellular components (CC) terms were related to GO: 0030667 (secretory granule membrane) and GO: 0001931 (uropod); and molecular functions (MF) terms were associated with GO: 0140375 (immune receptor activity) and GO: 0003735 (structural constituent of ribosome) (Supplementary Table 3). Then, we compared the differences in immune cell infiltration between the two immune patterns and discovered that immune cluster A had significantly higher levels of MDSC, neutrophil, plasmacytoid dendritic cell, and type 17 T helper cell expression than immune cluster B did (Figure 6(a)). The quantity of immune cells in IDD samples was then determined using ssGSEA, and the relationship between the 11 IRGs and immune cells was assessed (Figure 6(b)).

### 3.6. Identification of Two Distinct Gene Patterns

**Figure 7:**
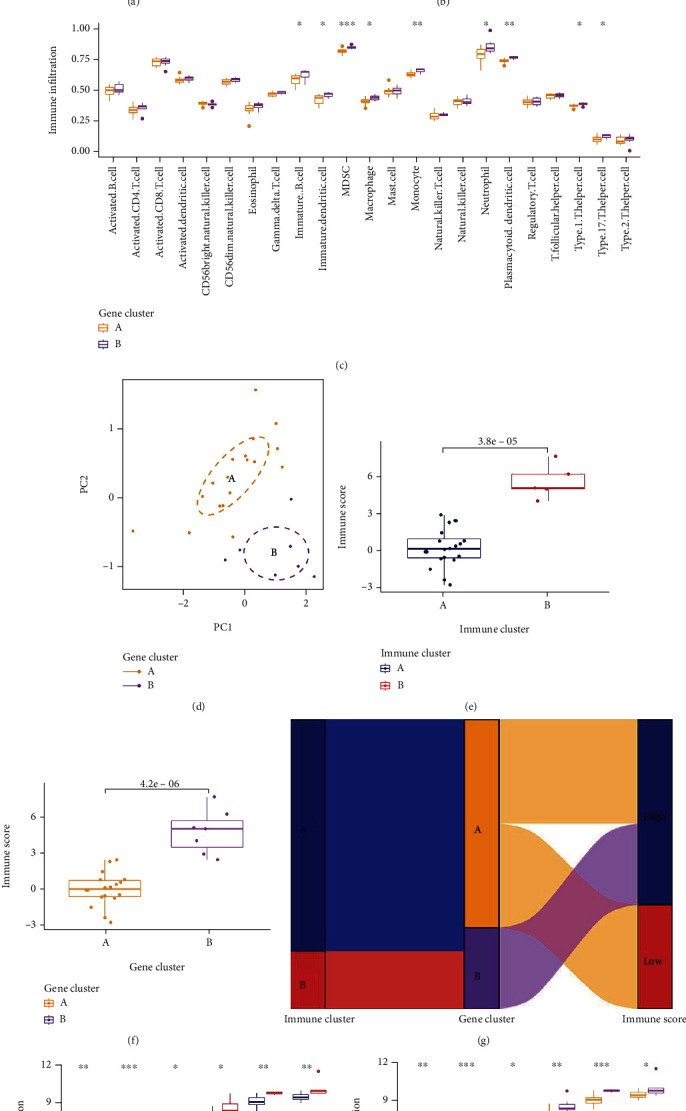
(a) Consensus matrices of the 220 DEGs. (b) Differential expression histogram of the 11 IEGs in gene cluster A and B. (c) Differential immune cell infiltration among gene cluster A and B. (d) PCA for gene cluster A and B. (e) Differences in immune score between immune cluster A and B. (f) Differences in immune score between gene cluster A and B. (g) The Sankey diagram of relationship between immune patterns, gene patterns, and immune scores. (h) Differential expression levels of the ADAMTS, ATG7, MMP13, NLRP3, TGFB1, and TLR4 among immune cluster A and B. (i) Differential expression levels of the ADAMTS, ATG7, MMP13, NLRP3, TGFB1, and TLR4 among gene cluster A and B.

The consensus clustering approach was applied to categorize the IDD patients into several genomic subtypes on the basis of the 220 DEGs in order to further confirm the immune patterns (Supplementary Table 4). Gene clusters A and B, which we discovered to be two gene patterns, matched the grouping of immune patterns (Figure 7(a) and Supplementary Figure 3).  Figure 7(b) displays the 11 IRGs in gene clusters A and B at various expression levels. The differential expression levels of the 11 IRGs and immune cell infiltration between gene clusters A and B were identical to those in the immune patterns, as shown in Figures 7(c) and 7(d). This again validates the accuracy of our grouping by the consensus clustering method. To quantify the gene patterns, we utilized PCA algorithms to calculate the immune score for each sample. We then compared the immune score between distinct immune patterns and gene patterns. The results showed that the immune score in immune cluster B or gene cluster B was higher (Figures 7(e) and 7(f) and Supplementary Table 5). The relationship between immune patterns, gene patterns, and immune scores was visualized in a Sankey diagram (Figure 7(g)).

### 3.7. Role of Immune Patterns in Distinguishing IDD

To further illuminate the association between different patterns and IDD, we evaluated the relationship between different patterns and these genes that have been demonstrated to be significantly associated with the development and progression of IDD, including ADAMTS, ATG7, MMP13, NLRP3, TGFB1, and TLR4. The expression levels of ATG7, NLRP3,TGFB1, and TLR4 were higher in immune cluster B and gene cluster B, indicating that immune cluster B and gene cluster B are highly associated with IDD having immune response characteristics (Figures 7(h) and 7(i)).

## 4. Discussion

IDD is a widespread degenerative disease that causes low back pain. There is increasing evidence that IRGs are involved in many biological processes. Usually, the normal nucleus is separated from the immune system through the peripheral intact structural disc structure, while after damage, the nucleus is exposed to the immune system, causing a range of autoimmune responses that play an important role in the progression of IDD [28]. In recent years, links between IDD and immune cells have been gradually revealed by researchers [29]. For example, T cells, B cells, and neutrophils may be associated with autoimmune responses triggered by exposure to the nuclear pulposus [30]. Studies have revealed sex-specific DNA methylation signatures in T cells that can distinguish chronic low back pain participants from healthy controls [31]. The latest study suggests that acute inflammatory response through neutrophil activation prevents the development of chronic pain [32]. Therefore, the purpose of our study was to explore the significance of IRGs in IDD.

We first recognized six IRGs through differential expression analysis between healthy and IDD samples. A RF model was developed to choose six IRGs (FPR1, RLN1, S100Z, IFNGR2, KLRK1, and CTSS) to predict the incidence of IDD. Nevertheless, owing to the lack of datasets containing IRGs in public databases, we could not validate our model in another independent dataset. A nomogram was developed on the basis of the six candidate IRGs, and the DCA curve demonstrated that decisions on the basis of the nomogram may benefit IDD patients.

During inflammation, FPR1 is highly expressed on activated neutrophils and monocytes/macrophages [33]. Xiao et al. used a molecular imaging modality targeting FPR1 to directly, noninvasively, and timely detect leukocyte infiltration around the site of acute intervertebral disc herniation. It has advanced our understanding of the etiology of IDD and has facilitated drug delivery and therapeutic monitoring of herniated discs [34]. Another study confirmed that a new FT-C 60 binds preferentially to FPR-1 on activated macrophages and significantly attenuates the mRNA expression of proinflammatory factors [35]. It has the potential for targeted therapy of degenerative disc disease. The role of RLN1 has not been demonstrated in IDD up to now, but Jin et al. predicted it as an immune-related biomarker of sciatica, which also falls within the category of IDD [36]. The calcium-binding protein S100A9 has been shown to induce nucleus cell apoptosis through the activation of the NF-B signaling pathway, cause matrix degradation and amplify inflammation, and can be used as a biomarker for IDD [37, 38]. However, S100Z, affiliated with the same family, has not been mentioned in the progression of IDD. IFNGR2 has not been shown to participate in the IDD process, but it has been found in rheumatoid arthritis [39]. However, in the process of IDD, the inflammatory response in the intervertebral disc cannot be ignored, which means that IFNGR2 may play a role in IDD, which deserves further confirmation. KLRK1, also known as NKG2D, is an activating receptor expressed by all NK cells and T cell subsets. It is the primary recognition receptor for detecting and eliminating transforming and infected cells and is involved in the development of multiple inflammatory diseases [40]. Unfortunately, it has a great correlation with immunity, but it has not been demonstrated in IDD. Similarly, the role of CTSS has not been demonstrated in IDD, but CTSS participates in the regulation of the immune microenvironment. CTSS inhibition can exert antitumor effects by boosting immune responses [41].

In our study, ADAMTS, ATG7, MMP13, NLRP3, TGFB1, and TLR4 could be used as potential immune-related biomarkers for IDD. ATG7, NLRP3, and TGFB1 and TLR4 expression were significantly higher in immune cluster B, which means that the IRG B gene cluster is highly associated with an IDD characterized by an immune response. Previous studies have confirmed that ATG7 was highly expressed in the normal disc, which inhibited NP cell apoptosis and maintained the degradation balance of extracellular matrix by activating autophagy, suggesting that the high expression of ATG7 in immune cluster B indicates a good prognosis [42, 43]. NLRP3 is thought to be a marker of pyrolysis and is associated with various chronic inflammatory diseases. Also in IDD, pyrolysis, as a form of cell death distinct from apoptosis, also exacerbated the degenerative progression. Silencing NLRP3 can decrease the activation of a range of markers associated with pyrolysis in IDD [44]. In other words, the high expression of NLRP3 in immune cluster B indicates a poor prognosis, suggesting that the IDD is in the process. TGFB1 is a multifunctional regulator of cellular activity. Studies confirmed that inhibition of TGFB1 expression significantly inhibited inflammation and low back pain in IDD [45]. In addition, inhibition of TGFB1 expression also promoted NP cell proliferation, reduced apoptosis, and remodelled the extracellular matrix [46]. Therefore, the high expression of TGFB1 in immune cluster B indicates a poor prognosis. Studies have confirmed that TLR4 was highly expressed in IDD tissues, which promoted the release of proinflammatory factors and increased apoptosis, excessive degradation of the extracellular matrix, low back pain, and so on [47, 48]. So the high expression of TLR4 in immune cluster B similarly suggests a poor prognosis.

Nevertheless, there are still some limitations to our study. On one hand, the study is based on data analysis of public data, and the results obtained should be validated by in vivo or in vitro experiments. On the other hand, the sample size included was too small due to the lack of data sets. Therefore, in future studies, we will continue to expand the sample size and perform basic experiments to validate our study so that it can be better applied in the clinical situation.

## 5. Conclusion

Overall, our study constructed a nomogram that can precisely predict the prevalence of IDD and identified two different immune patterns, providing a novel perspective on IDD prevention and diagnosis.

## Data Availability

The data used in this study are available from the corresponding authors upon request.

## Supplementary Materials

Supplementary Materials
*Supplementary 1*. Supplementary Figure 1: the AUC values of the ROC curve of CTSS, FPR1, IFNGR2, KLRK1, RLN1, and S100Z. *Supplementary 2*. Supplementary Figure 2: (a–g) Consensus matrices of the 11 IRGs for *k* = 3 − 9. *Supplementary 3*. Supplementary Figure 3: (a–g) Consensus matrices of the 220 DEGs for *k* = 3 − 9. *Supplementary 4*. Supplementary Table 1: the list of IRGs. *Supplementary 5*. Supplementary Table 2: the expression profiles of differentially expressed IRGs. *Supplementary 6*. Supplementary Table 3: GO enrichment analysis of DEGs. *Supplementary 7*. Supplementary Table 4: the DEGs. *Supplementary 8*. Supplementary Table 5: immune score for each sample.
