# Supplementary material for: Significance of Immune-Related Genes in the Diagnosis and Classification of Intervertebral Disc Degeneration
Source: J Immunol Res. 2022 Aug 30;2022:2616260. doi: 10.1155/2022/2616260 (PMC9448583; doi:10.1155/2022/2616260)
Supplement: Supplementary Materials — Supplementary 1. Supplementary Figure 1: the AUC values of the ROC curve of CTSS, FPR1, IFNGR2, KLRK1, RLN1, and S100Z. Supplementary 2. Supplementary Figure 2: (a–g) Consensus matrices of the 11 IRGs for k = 3 − 9. Supplementary 3. Supplementary Figure 3: (a–g) Consensus matrices of the 220 DEGs for k = 3 − 9. Supplementary 4. Supplementary Table 1: the list of IRGs. Supplementary 5. Supplementary Table 2: the expression profiles of differentially expressed IRGs. Supplementary 6. Supplementary Table 3: GO enrichment analysis of DEGs. Supplementary 7. Supplementary Table 4: the DEGs. Supplementary 8. Supplementary Table 5: immune score for each sample. [file 2616260.f1.pdf]

Supplementary Figure 1: The AUC values of the ROC curve of CTSS, FPR1, IFNGR2, KLRK1,RLN1 and S100Z.

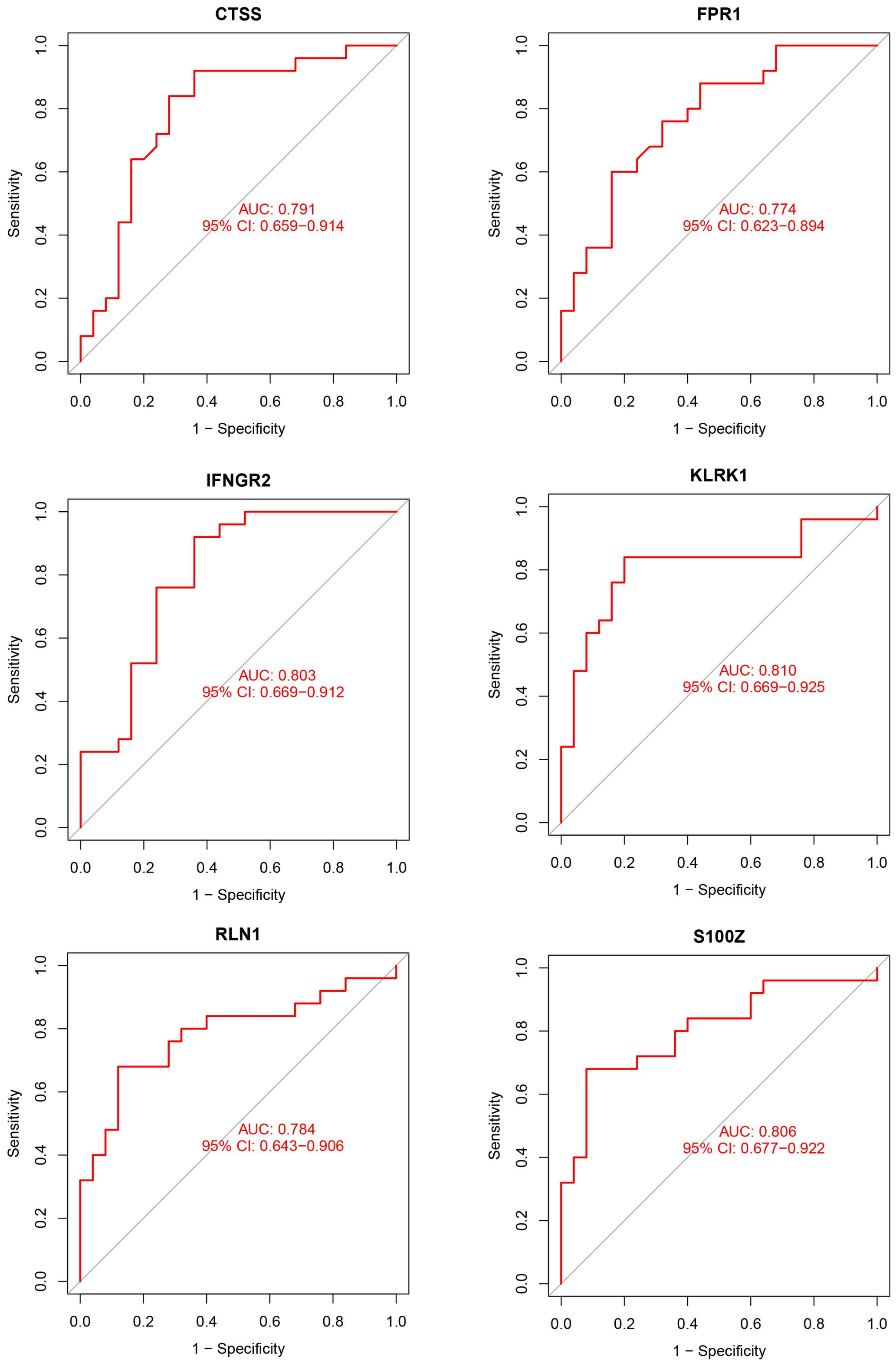

Supplementary Figure 2: (A-G) Consensus matrices of the 11 IRGs for k = 3-9.

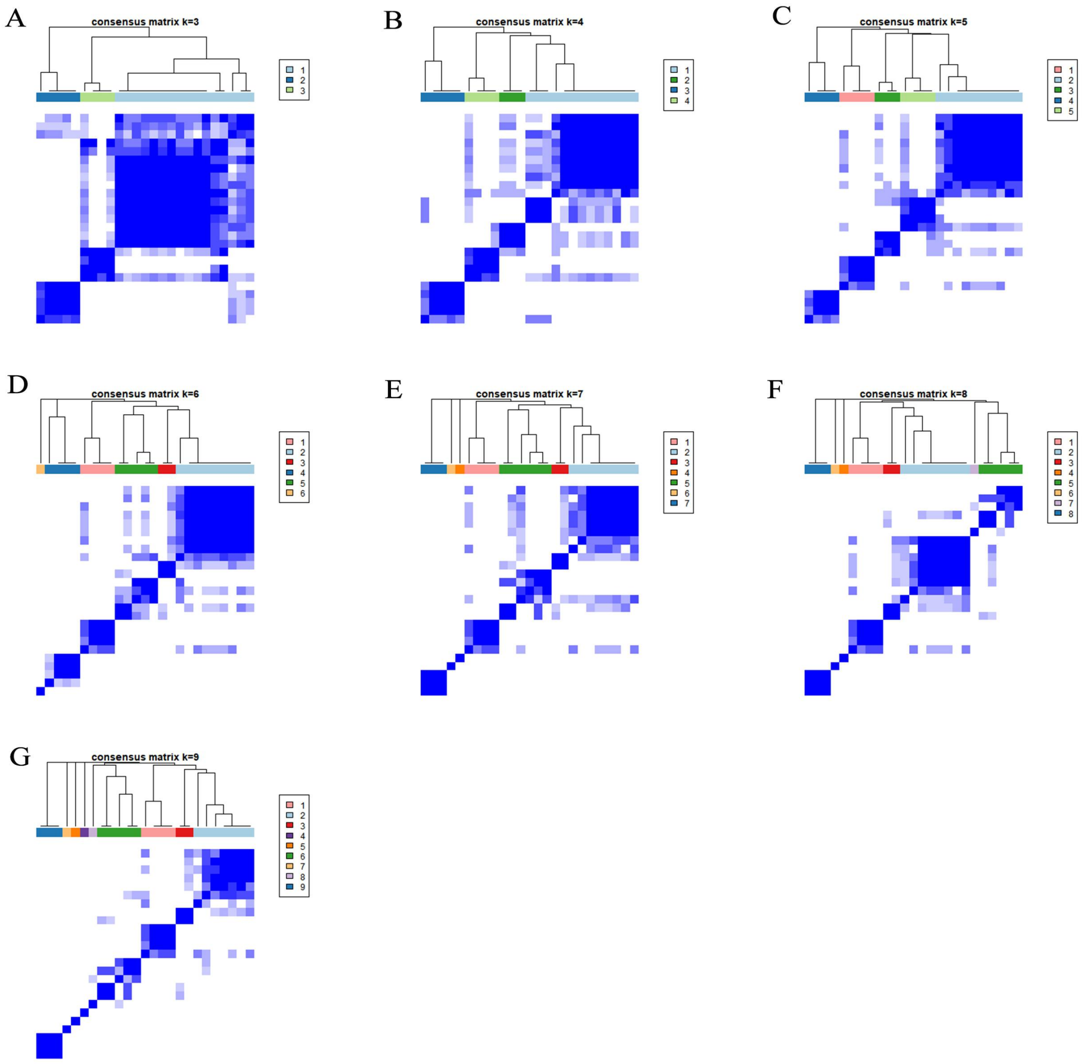

Supplementary Figure 3: (A-G) Consensus matrices of the 220 DEGs for k = 3-9.

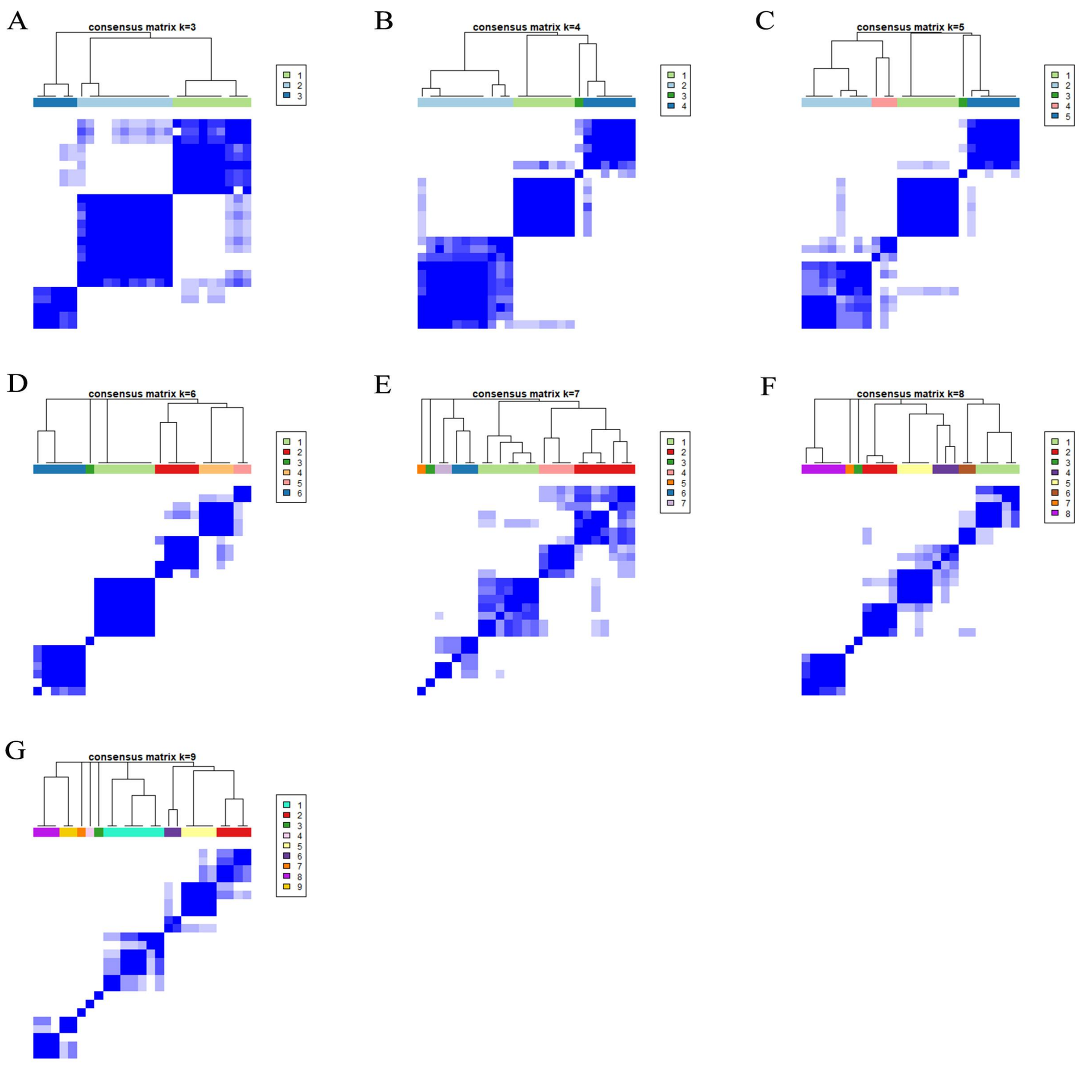

Supplementary Table 1: The list of IRGs.

AZGP1
B2M
CALR
CANX
CD1A
CD1B
CD1C
CD1D
CD1E
CD4
CD8A
CD8B

CD74
CREB1
CTSB
CTSE
CTSL
CTSS
FCER1G
FCGRT
PDIA3
HFE
HLA-A
HLA-B
HLA-C
HLA-DMA
HLA-DMB
HLA-DOA
HLA-DOB
HLA-DPA1
HLA-DPB1
HLA-DQA1
HLA-DQA2
HLA-DQB1
HLA-DRA
HLA-DRB1
HLA-DRB3
HLA-DRB4
HLA-DRB5
HLA-E
HLA-F
HLA-G
HLA-H
MR1
HSPA1A
HSPA1B
HSPA1L
HSPA2
HSPA4
HSPA5
HSPA6
HSPA8
HSP90AA1
HSP90AB1
ICAM1
IFNA1

IFNA2
IFNA4
IFNA5
IFNA6
IFNA7
IFNA8
IFNA10
IFNA13
IFNA14
IFNA16
IFNA17
IFNA21
IFNG
KIR2DL1
KIR2DL2
KIR2DL3
KIR2DL4
KIR2DS1
KIR2DS3
KIR2DS4
KIR2DS5
KIR3DL1
KIR3DL2
KLRC1
KLRC2
KLRC3
KLRD1
LTA
CIITA
MICA
MICB
NFYA
NFYB
NFYC
LGMN
PSMB8
PSMC1
PSMC2
PSMC3
PSMC4
PSMC5
PSMC6
PSMD1
PSMD2

PSMD3
PSMD4
PSMD5
PSMD7
PSMD8
PSMD10
PSMD11
PSMD13
PSME1
PSME1
PSME2
PSME2
RELB
RFX5
RFXAP
SLC10A2
TAP1
TAP2
TAPBP
THBS1
SEM1
KLRC4
AP3B1
RFXANK
PSMD6
PSME3
PSMD14
CLEC4M
IFI30
PROCR
ADRM1
ECPAS
TRPC4AP
CD209
UBXN1
ERAP1
TAPBPL
KIR2DL5A
ERAP2
ULBP3
ULBP2
ULBP1
KIR3DL3
RAET1E

RAET1L
UBR1
RAET1G
PDIA2
HAMP
PI3
CAMP
DEFB4A
PPBP
REG3G
CXCL14
CXCL16
SLPI
CXCL8
CXCL10
CXCL9
CXCL5
CXCL11
CXCL6
CXCL1
CXCL12
CXCL13
CXCL2
PF4
XCL1
CXCL3
DEFB103B
CCL13
CCL1
DEFB1
CCL8
ELANE
DEFB103A
DEFA3
DEFA1
TMSB10
DEFA6
DEFA5
DEFA4
LCN2
LCN1
COLEC10
BPI
S100A9

S100A8
DCD
LCN6
S100A12
HTN3
LCN8
DEFA1B
CCR10
CELA1
DEFB106A
PENK
BPIFC
MMP12
BPIFB6
LEAP2
SFTPD
LCN9
BPIFB2
PTGDS
TMSB4X
PGLYRP1
ZC3HAV1
TMSB15A
S100B
S100A13
S100A6
DEFB119
DEFB107A
DEFB105A
SERPIND1
DEFB129
DEFB127
S100P
S100A7
DEFB104A
DEFB126
DEFB106B
DEFB104B
DEFB107B
PGLYRP3
PGLYRP2
S100A10
S100A2
DEFB125

DEFB123
DEFB105B
DEFB132
BPIFB3
LCN12
PGLYRP4
S100A11
S100A5
S100A3
S100A1
DEFB128
DEFB108B
HTN1
LMBR1L
S100A7A
DEFB118
COLEC12
TMSB4Y
DEFB131A
DEFB134
DEFB130A
DEFB124
DEFB121
DEFB116
DEFB115
DEFB114
DEFB113
DEFB112
DEFB110
TMSB15B
DEFB133
S100Z
MAVS
TMSB4XP8
S100A14
LCN10
S100A16
DEFB136
DEFB135
DEFB117
DEFB110
ZC3HAV1L
S100A7L2
MBL3P

DEFB4B
BPIFB4
IFNAR1
AZU1
DEFB131B
DEFA1A3
LCN1P1
S100G
DEFA7P
DEFB130B
DEFB108F
DEFB131C
TCHHL1
TINAGL1
IFNGR1
SLC22A17
WFIKKN1
WFDC2
IL6
UMODL1
TGFB1
PF4V1
MMP9
ANOS1
TLR4
IFNG
SPAG11B
A2M
CTSL
NFKB1
APOBEC3G
FABP6
NOD2
MBL2
SFTPA1
RBP1
TLR2
SLC40A1
PLAU
IL1B
PAEP
HJV
MUC5AC
CTSS

OBP2A
PLTP
MX1
DDX58
IFNL1
IRF3
SFTPA2
LPA
LBP
RBP4
SFTPA1
NOX4
LTF
IFNB1
RBP5
FABP7
FABP5
FABP3
FABP2
FABP4
R3HDML
BPIFA3
BPIFB1
OASL
CRABP2
CRABP1
RBP7
DUOX1
OBP2B
RBP2
LCN15
CETP
FABP12
FABP9
BPIFA1
LCNL1
C8G
SPAG11A
PI15
NOX1
PMP2
APOD
ORM2
ORM1

TNF
CTSG
PRTN3
MAPK1
PML
AEN
CYBB
BPIFA2
ISG20
BCL3
ISG20L2
NOX5
NOX3
DUOX2
TLR3
TFRC
IFIH1
LRP1
TRIM5
IDO1
GDF15
NEDD4
ADIPOQ
STAT3
STAT1
IFNL2
SOCS3
SEMG1
TNFSF10
CCL20
SOCS1
RNASEL
IRF1
IL15
APOBEC3F
PLAAT4
CHIT1
IFNA1
CD40
TLR7
PPIA
HFE
ZYX
NLRX1

PGC
VEGFA
IKBKE
ISG15
DHX58
TNFAIP3
TFR2
FCN2
MUC4
F2R
ELN
IL27
MAPT
LYZ
CCL5
LEP
CYLD
KLKB1
CST4
CSRP1
MAPK14
JUN
ITGAV
IRF5
CCR6
IL12B
TLR8
GNLY
CD81
EIF2AK2
APOM
CACYBP
NOD1
MAPK8
MAPK3
BST2
BPHL
PLA2G2A
GRN
NEWENTRY
PDGFRA
GNAI1
WNT5A
FURIN

ADAR
TYK2
NOS2
TRAF3
TPT1
TPM2
NEO1
AHNAK
TLR1
TK2
PRDX2
MX2
FGF2
FGA
TCF7L2
F2RL1
TKFC
MSR1
NFKBIZ
LMBR1
EPPIN
SRC
MPO
ELAVL1
ROBO3
SP1
SOD1
PDF
DLL4
ECD
SLC11A1
DMBT1
STING1
SKIV2L
SEMG2
LTA
DES
DCK
DAXX
TNFRSF10A
TNFRSF10B
EED
CCL4
LIMS1

LALBA
APOBEC3H
TMPRSS6
SPINK5
MARCO
BECN1
TNFSF11
KNG1
CSK
KLRK1
KCNH2
JUND
JAK1
CREB1
CLDN4
CCL28
RNASE3
RN7SL1
IRF7
IREB2
ILK
IL18
IL17A
LTB4R
APOBEC3A
MASP2
TRIM27
RELA
IL7R
IL1A
PTX3
IFNAR2
IFN1@
SYTL1
APOBEC3C
DDX17
PTGS2
HTR1A
SEPTIN7
CD40LG
CD14
CD8A
CD4
MASP1

PROC
MAP2K2
MAP2K1
HRG
NDRG1
IRF9
TRIM22
LANCL1
PPP4C
HMOX1
HMGB1
HLA-B
RNASE7
ABCC4
HGF
HDAC1
IFNLR1
PLSCR1
B2M
BACH2
TANK
PIK3CG
ARRB1
RSAD2
STAB2
TBK1
PDYN
PDGFRB
PDCD1
PCSK2
PCSK1
ARG2
AQP9
FASLG
APOH
BIRC5
ANXA6
IL22
VTN
VIM
VCAM1
PRDX1
GFAP
GBP2

ALB
SLC29A3
OAS1
AGER
UNC93B1
TNFSF4
NOS1
ACTG1
ACTA1
ACO1
SERPINA3
CXCR1
CCL15
CCL14
CCL4
CCL16
CCL19
CCL13
CCL18
CCL17
CCL26
CCL22
CCR3
CCL28
CCL4L1
ACKR2
CCR7
CCL27
CCR8
ACKR4
CCR10
CCL2
CCL21
CCL7
CCL5
CCL3
CCL20
CCL11
CCR5
CCL23
CCL25
CCL1
CCL3L3
CCL4L2

CXCL12
XCL1
CCL8
CCL3L1
CCR1
CCL24
XCL2
CXCL1
CXCL10
CXCR4
CXCL2
CXCR6
CCR4
CXCL11
TAFA5
TAFA3
TAFA4
TAFA1
TAFA2
CCL15-CCL14
IL6
TNF
IL1B
IL18
PTK2B
VEGFA
IL4
CDH1
CD40
DEFB103B
F2RL1
MMP9
LTBP1
DEFB4A
TNFSF10
IL13
IL10
IL2
PPARG
FGR
MIF
CRP
JAK2
IL1A

PTK2
PTGDR
CD86
HCK
ARRB1
GNAI1
VDR
OLR1
GRK2
TXK
RNASE2
CD79A
CD79B
LYN
SYK
BTK
BLNK
VAV3
VAV1
VAV2
RAC1
RAC2
RAC3
PPP3CA
PPP3CB
PPP3CC
CHP1
PPP3R1
PPP3R2
CHP2
NFAT5
NFATC1
NFATC2
NFATC3
NFATC4
HRAS
KRAS
NRAS
FOS
JUN
CARD11
BCL10
MALT1
CHUK

IKBKB
IKBKG
NFKB1
RELA
NFKBIA
NFKBIB
NFKBIE
CD81
CD19
CR2
PIK3R5
PIK3R1
PIK3R2
PIK3R3
PIK3CA
PIK3CB
PIK3CD
PIK3CG
AKT3
AKT1
AKT2
GSK3B
INPP5D
CD22
CD72
PTPN6
LILRB3
FCGR2B
RASGRP3
PLCG2
PRKCB
IFITM1
IGH
IGHA1
IGHA2
IGHD
IGHD1-1
IGHD1-14
IGHD1-20
IGHD1-26
IGHD1-7
IGHD2-15
IGHD2-2
IGHD2-21

IGHD2-8
IGHD3-10
IGHD3-16
IGHD3-22
IGHD3-3
IGHD3-9
IGHD4-11
IGHD4-17
IGHD4-23
IGHD4-4
IGHD5-12
IGHD5-18
IGHD5-24
IGHD5-5
IGHD6-13
IGHD6-19
IGHD6-25
IGHD6-6
IGHD7-27
IGHE
IGHG1
IGHG2
IGHG3
IGHG4
IGHJ1
IGHJ2
IGHJ3
IGHJ4
IGHJ5
IGHJ6
IGHM
IGH
IGHV1-18
IGHV1-2
IGHV1-24
IGHV1-3
IGHV1-45
IGHV1-46
IGHV1-58
IGHV1-69
IGHV1-8
IGHV1-38-4
IGHV1-69-2
IGHV2-26

IGHV2-5
IGHV2-70
IGHV3-11
IGHV3-13
IGHV3-15
IGHV3-16
IGHV3-20
IGHV3-21
IGHV3-23
IGHV3-30
IGHV3-30-3
IGHV3-30-5
IGHV3-33
IGHV3-35
IGHV3-38
IGHV3-43
IGHV3-48
IGHV3-49
IGHV3-53
IGHV3-64
IGHV3-66
IGHV3-7
IGHV3-72
IGHV3-73
IGHV3-74
IGHV3-9
IGHV3-38-3
IGHV3-69-1
IGHV4-28
IGHV4-30-1
IGHV4-30-2
IGHV4-30-4
IGHV4-31
IGHV4-34
IGHV4-39
IGHV4-4
IGHV4-59
IGHV4-61
IGHV4-38-2
IGHV5-51
IGHV5-10-1
IGHV6-1
IGHV7-4-1
IGHV7-81

IGK
IGKC
IGKDEL
IGKJ
IGKJ1
IGKJ2
IGKJ3
IGKJ4
IGKJ5
IGKV@
IGKV1-12
IGKV1-13
IGKV1-16
IGKV1-17
IGKV1-27
IGKV1-33
IGKV1-37
IGKV1-39
IGKV1-5
IGKV1-6
IGKV1-8
IGKV1-9
IGKV1D-12
IGKV1D-13
IGKV1D-16
IGKV1D-17
IGKV1D-33
IGKV1D-37
IGKV1D-39
IGKV1D-42
IGKV1D-43
IGKV1D-8
IGKV2-24
IGKV2-28
IGKV2-30
IGKV2-40
IGKV2D-24
IGKV2D-28
IGKV2D-29
IGKV2D-30
IGKV2D-40
IGKV3-11
IGKV3-15
IGKV3-20

IGKV3-7
IGKV3D-11
IGKV3D-15
IGKV3D-20
IGKV3D-7
IGKV4-1
IGKV5-2
IGKV6-21
IGKV6D-21
IGKV6D-41
IGL
IGLC1
IGLC2
IGLC3
IGLC6
IGLC7
IGLJ
IGLJ1
IGLJ2
IGLJ3
IGLJ4
IGLJ5
IGLJ6
IGLJ7
IGLV@
IGLV1-36
IGLV1-40
IGLV1-44
IGLV1-47
IGLV1-50
IGLV1-51
IGLV10-54
IGLV11-55
IGLV2-11
IGLV2-14
IGLV2-18
IGLV2-23
IGLV2-33
IGLV2-8
IGLV3-1
IGLV3-10
IGLV3-12
IGLV3-16
IGLV3-19

IGLV3-21
IGLV3-22
IGLV3-25
IGLV3-27
IGLV3-32
IGLV3-9
IGLV4-3
IGLV4-60
IGLV4-69
IGLV5-37
IGLV5-39
IGLV5-45
IGLV5-48
IGLV5-52
IGLV6-57
IGLV7-43
IGLV7-46
IGLV8-61
IGLV9-49
C3
C5
CAMP
CCL1
CCL11
CCL13
CCL14
CCL15-CCL14
CCL15
CCL16
CCL17
CCL18
CCL19
CCL2
CCL20
CCL21
CCL22
CCL23
CCL24
CCL25
CCL26
CCL27
CCL28
CCL3
CCL3L1

CCL3P1
CCL3L3
CCL4
CCL4L2
CCL4L1
CCL5
CCL7
CCL8
CKLF
CMA1
CTSG
CX3CL1
CXCL1
CXCL10
CXCL11
CXCL12
CXCL13
CXCL14
CXCL16
CXCL17
CXCL2
CXCL3
CXCL5
CXCL6
CXCL9
CCN1
DEFA1
DEFA3
DEFA5
DEFB1
DEFB103B
DEFB104A
DEFB4A
EDN1
EDN2
EDN3
FGF10
FGF2
HTN3
CXCL8
LECT2
PF4
PF4V1
PLAU

PPBP
PPBPP1
PROK2
RNASE2
SAA1
SAA2
SBDS
SEMA3A
SEMA3B
SEMA3C
SEMA3D
SEMA3E
SEMA3F
SEMA3G
SEMA4A
SEMA4B
SEMA4C
SEMA4D
SEMA4F
SEMA4G
SEMA5A
SEMA5B
SEMA6A
SEMA6B
SEMA6C
SEMA6D
SEMA7A
SLIT1
SLIT2
TNC
TYMP
XCL1
XCL2
C5AR1
ACKR2
CCR1
CCR10
CCR3
CCR4
CCR5
CCR6
CCR7
CCR8
CCR9

ACKR4
CCRL2
CMKLR1
CX3CR1
CXCR3
CXCR4
CXCR5
CXCR6
ACKR3
CYSLTR1
CYSLTR2
ACKR1
EDNRA
EDNRB
FPR1
FPR2
FPR2
GPR17
GPR32
GPR33
PTGDR2
C5AR2
CXCR1
CXCR2
LTB4R
LTB4R2
PLAUR
PLXNA1
PLXNA2
PLXNA3
PLXNA4
PLXNB1
PLXNB2
PLXNB3
PLXNC1
PLXND1
PTAFR
ROBO1
ROBO2
ROBO3
RXFP3
XCR1
ADIPOQ
ADM

ADM2
AGRP
AGT
AMBN
AMELX
AMH
ANGPTL5
ANGPTL7
APLN
AREG
MANF
CDNF
ARTN
AVP
AZU1
BDNF
BMP1
BMP10
BMP15
BMP2
BMP3
BMP4
BMP5
BMP6
BMP7
BMP8A
BMP8B
BTC
MYDGF
C3
C5
CALCA
CALCB
CAMP
CAT
CCK
CCL1
CCL11
CCL13
CCL14
CCL15-CCL14
CCL15
CCL16
CCL17

CCL18
CCL19
CCL2
CCL20
CCL21
CCL22
CCL23
CCL24
CCL25
CCL26
CCL27
CCL28
CCL3
CCL3L1
CCL3P1
CCL3L3
CCL4
CCL4L2
CCL4L1
CCL5
CCL7
CCL8
CD320
CD40LG
CD70
ADA2
CER1
CGA
CGB3
CGB1
CGB2
CGB5
CGB7
CGB8
CHGA
CHGB
CKLF
CLCF1
CLEC11A
CMA1
CMTM1
CMTM2
CMTM3
CMTM4

CMTM5
CMTM6
CMTM7
CMTM8
CNTF
CORT
CRH
CSF1
CSF2
CSF3
CSH1
CSH2
CSHL1
CSPG5
CTF1
CCN2
CTSG
CX3CL1
CXCL1
CXCL10
CXCL11
CXCL12
CXCL13
CXCL14
CXCL16
CXCL17
CXCL2
CXCL3
CXCL5
CXCL6
CXCL9
CCN1
DEFA1
DEFA3
DEFA5
DEFB1
DEFB103B
DEFB104A
DEFB4A
DKK1
EBI3
EDN1
EDN2
EDN3

EGF
EPGN
EPO
EREG
ESM1
FAM3B
FAM3C
FAM3D
FASLG
FGF1
FGF10
FGF11
FGF12
FGF13
FGF14
FGF16
FGF17
FGF18
FGF19
FGF2
FGF20
FGF21
FGF22
FGF23
FGF3
FGF4
FGF5
FGF6
FGF7
FGF8
FGF9
VEGFD
FIGNL2
FLT3LG
FSHB
GAL
GALP
GAST
GCG
GDF1
GDF10
GDF11
GDF15
GDF2

GDF3
GDF5
GDF6
GDF7
GDF9
GDNF
GH1
GH2
GHRH
GHRL
GIP
GKN1
GMFB
GMFG
GNRH1
GNRH2
GPHA2
GPHB5
GPI
GREM1
GREM2
GRN
GRP
GUCA2A
HAMP
HBEGF
HDGF
HDGFL3
HGF
HTN3
IAPP
IFNA1
IFNA10
IFNA13
IFNA14
IFNA16
IFNA17
IFNA2
IFNA21
IFNA4
IFNA5
IFNA6
IFNA7
IFNA8

IFNB1
IFNE
IFNG
IFNK
IFNW1
IGF1
IGF2
IL10
IL11
IL12A
IL12B
IL13
IL15
IL16
IL17A
IL17B
IL17C
IL17D
IL17F
IL18
IL19
IL1A
IL1B
IL1F10
IL36RN
IL36A
IL37
IL36B
IL36G
IL1RN
IL2
IL20
IL21
IL22
IL23A
IL24
IL25
IL26
IL27
IFNL2
IFNL3
IFNL1
IL3
IL31

IL32
IL33
IL34
IL4
IL5
IL6
IL6ST
IL7
CXCL8
IL9
INHA
INHBA
INHBB
INHBC
INHBE
INS
INS-IGF2
INSL3
INSL4
INSL5
INSL6
JAG1
JAG2
FGF7P6
FGF7P3
KITLG
KL
LACRT
LECT2
LEFTY1
LEFTY2
LEP
LHB
LIF
LRSAM1
LTA
LTB
LTBP1
LTBP2
LTBP3
LTBP4
MDK
MIA
MIF

MLN
MSTN
NAMPT
NDP
NENF
NGF
NMB
NODAL
CCN3
NPFF
NPPA
NPPB
NPPC
NPY
NRG1
NRG2
NRG3
NRG4
NRTN
NTF3
NTF4
NTS
NUDT6
OGN
OSGIN1
OSM
OSTN
OXT
ENDOU
PDGFA
PDGFB
PDGFC
PDGFD
PDGFRA
PDGFRB
PDGFRL
PDYN
PENK
PF4
PF4V1
PGF
PLAU
PMCH
PNOC

POMC
PPBP
PPBPP1
PPBPP2
PPY
PRL
PRLH
PROK1
PROK2
PSPN
PTH
PTH2
PTHLH
PTN
PYY
QRFP
RABEP1
RABEP2
REG1A
RETN
RETNLB
RLN1
RLN2
RLN3
RNASE2
S100A6
SAA1
SAA2
SBDS
SCG2
SCGB3A1
SCT
AIMP1
SECTM1
SEMA3A
SEMA3B
SEMA3C
SEMA3D
SEMA3E
SEMA3F
SEMA3G
SEMA4A
SEMA4B
SEMA4C

SEMA4D
SEMA4F
SEMA4G
SEMA5A
SEMA5B
SEMA6A
SEMA6B
SEMA6C
SEMA6D
SEMA7A
SLIT1
SLIT2
SLURP1
SPP1
SST
STC1
STC2
TAC1
TDGF1
TDGF1P3
TG
TGFA
TGFB1
TGFB2
TGFB3
THPO
TNC
TNF
TNFRSF11B
TNFSF10
TNFSF11
TNFSF12
TNFSF13
TNFSF13B
TNFSF14
TNFSF15
TNFSF18
TNFSF4
TNFSF8
TNFSF9
TOR2A
TRH
TSHB
TSLP

TXLNA
TYMP
UCN
UCN2
UCN3
UTS2
UTS2B
VEGFA
VEGFB
VEGFC
VGF
VIP
XCL1
XCL2
ACVR1B
ACVR1C
ACVR2A
ACVR2B
ACVRL1
ADCYAP1R1
ADIPOR1
ADIPOR2
ADRB1
ADRB2
AGTR1
AGTR2
AMHR2
ANGPT1
ANGPT4
ANGPTL1
ANGPTL2
ANGPTL3
ANGPTL4
ANGPTL6
APLNR
AR
AVPR1A
AVPR1B
AVPR2
BMPR1A
BMPR1B
BMPR2
BRD8
C3AR1

C5AR1
CALCR
CALCRL
ACKR2
CCR1
CCR10
CCR3
CCR4
CCR5
CCR6
CCR7
CCR8
CCR9
ACKR4
CCRL2
CD40
CMKLR1
CNTFR
CRHR1
CRHR2
CRIM1
CRLF1
CRLF2
CRLF3
CSF1R
CSF2RA
CSF2RB
CSF3R
CX3CR1
CXCR3
CXCR4
CXCR5
CXCR6
ACKR3
CYSLTR1
CYSLTR2
ACKR1
EDNRA
EDNRB
EGFR
ENG
EPOR
ESR1
ESR2

ESRRA
ESRRB
ESRRG
FGFR1
FGFR2
FGFR3
FGFR4
FGFRL1
FLT1
FLT3
FLT4
FPR1
FPR2
FPR2
FSHR
GALR2
GALR3
GCGR
GHR
GHRHR
GHSR
GIPR
GLP1R
GLP2R
GNRHR
GPER1
GPR17
GPR32
GPR33
PTGDR2
C5AR2
HNF4A
HNF4G
HTR3A
HTR3B
HTR3C
HTR3D
HTR3E
IFNAR1
IFNAR2
IFNGR1
IFNGR2
IGF1R
IGF2R

IL10RA
IL10RB
IL11RA
IL12RB1
IL12RB2
IL13RA1
IL13RA2
IL15RA
IL2RB
IL17RA
IL17RB
IL17RC
IL17RD
IL17RE
IL18R1
IL18RAP
IL1R1
IL1R2
IL1RAP
IL1RL1
IL1RL2
IL20RA
IL20RB
IL21R
IL22RA1
IL22RA2
IL23R
IL27RA
IFNLR1
IL2RA
IL2RB
IL2RG
IL31RA
IL3RA
IL4R
IL5RA
IL6R
IL7R
CXCR1
CXCR2
IL9R
INSR
KDR
LEPR

LGR4
LGR5
LGR6
LHCGR
LIFR
LTB4R
LTB4R2
LTBR
MC1R
MC2R
MC3R
MC4R
MCHR1
MCHR2
MET
MLNR
MPL
MTNR1A
MTNR1B
NGFR
NMBR
NPR1
NPR3
NR0B1
NR0B2
NR1D1
NR1D2
NR1H2
NR1H3
NR1H4
NR1I2
NR1I3
NR2C1
NR2C2
NR2E1
NR2E3
NR2F1
NR2F2
NR2F6
NR3C1
NR3C2
NR4A1
NR4A2
NR4A3

NR5A1
NR5A2
NR6A1
NRP1
NRP2
OGFR
OPRD1
OPRK1
OPRL1
OPRM1
OSMR
OXTR
PGR
PGRMC2
PLAUR
PLXNA1
PLXNA2
PLXNA3
PLXNA4
PLXNB1
PLXNB2
PLXNB3
PLXNC1
PLXND1
PPARA
PPARD
PPARG
PRLHR
PRLR
PTAFR
PTGDR
PTGDS
PTGER1
PTGER2
PTGER3
PTGER4
PTGFR
PTH1R
PTH2R
RARA
RARB
RARG
ROBO1
ROBO2

ROBO3
RORA
RORB
RORC
RXFP1
RXFP2
RXFP3
RXRA
RXRB
RXRG
S1PR1
S1PR2
SCTR
SDC1
SDC2
SDC3
SDC4
SORT1
SSTR1
SSTR2
SSTR5
ST2
TACR1
TEK
TGFBR1
TGFBR2
TGFBR3
THRA
THRB
TIE1
TNFRSF10A
TNFRSF10B
TNFRSF10C
TNFRSF10D
TNFRSF11A
TNFRSF12A
TNFRSF13B
TNFRSF13C
TNFRSF14
TNFRSF17
TNFRSF18
TNFRSF19
TNFRSF1A
TNFRSF1B

TNFRSF21
TNFRSF25
TNFRSF4
TNFRSF6B
TNFRSF8
TNFRSF9
TRHR
TSHR
TUBB3
VDR
VIPR1
VIPR2
XCR1
IFNA10
IFNA13
IFNA14
IFNA16
IFNA17
IFNA2
IFNA21
IFNA4
IFNA5
IFNA6
IFNA7
IFNA8
IFNB1
IFNE
IFNG
IFNK
IFNW1
IFNAR2
IFNGR1
IFNGR2
IL11
IL12A
IL12B
IL13
IL15
IL16
IL17A
IL17B
IL17C
IL17D
IL17F

IL18
IL19
IL1A
IL1B
IL1F10
IL36RN
IL36A
IL37
IL36B
IL36G
IL1RN
IL2
IL20
IL21
IL22
IL23A
IL24
IL25
IL26
IL27
IFNL2
IFNL3
IFNL1
IL3
IL31
IL32
IL33
IL34
IL4
IL5
IL6
IL6ST
IL7
CXCL8
IL9
TXLNA
IL10RA
IL10RB
IL11RA
IL12RB1
IL12RB2
IL13RA1
IL13RA2
IL15RA

IL2RB
IL17RA
IL17RB
IL17RC
IL17RD
IL17RE
IL18R1
IL18RAP
IL1R1
IL1R2
IL1RAP
IL1RL1
IL1RL2
IL20RA
IL20RB
IL21R
IL22RA1
IL22RA2
IL23R
IL27RA
IFNLR1
IL2RA
IL2RB
IL2RG
IL31RA
IL3RA
IL4R
IL5RA
IL6R
IL7R
CXCR1
CXCR2
IL9R
ST2
HLA-A
HLA-B
HLA-C
HLA-E
HLA-G
KIR3DL1
KIR3DL2
KIR2DL1
KIR2DL2
KIR2DL3

KIR2DL4
KIR2DL5A
KLRC1
KLRC2
KLRC3
KLRD1
PTPN6
PTPN11
ICAM1
ICAM2
ITGAL
ITGB2
PTK2B
VAV3
VAV1
VAV2
RAC1
RAC2
RAC3
PAK1
MAP2K1
MAP2K2
MAPK1
MAPK3
TNF
CSF2
IFNG
KIR2DS1
KIR2DS3
KIR2DS4
KIR2DS5
NCR2
TYROBP
LCK
FCGR3A
FCGR3B
NCR1
NCR3
FCER1G
CD247
ZAP70
SYK
LCP2
LAT

PLCG1
PLCG2
SH3BP2
PIK3CA
PIK3CB
PIK3CD
PIK3CG
PIK3R5
PIK3R1
PIK3R2
PIK3R3
FYN
SHC2
SHC4
SHC3
SHC1
GRB2
SOS1
SOS2
HRAS
KRAS
NRAS
ARAF
BRAF
RAF1
MICA
MICB
ULBP3
ULBP2
ULBP1
KLRK1
HCST
CD48
CD244
PPP3CA
PPP3CB
PPP3CC
CHP1
PPP3R1
PPP3R2
CHP2
NFAT5
NFATC1
NFATC2

NFATC3
NFATC4
PRKCA
PRKCB
PRKCG
SH2D1B
SH2D1A
IFNGR1
IFNGR2
IFNA1
IFNA2
IFNA4
IFNA5
IFNA6
IFNA7
IFNA8
IFNA10
IFNA13
IFNA14
IFNA16
IFNA17
IFNA21
IFNB1
IFNAR1
IFNAR2
TNFSF10
TNFRSF10D
TNFRSF10C
TNFRSF10B
TNFRSF10A
FASLG
FAS
GZMB
PRF1
CASP3
BID
CD3D
CD3E
CD3G
CD247
CD4
CD8A
CD8B
PTPRC

LCK
FYN
ZAP70
LCP2
LAT
ITK
TEC
NCK1
NCK2
VAV3
VAV1
VAV2
GRAP2
GRB2
PAK1
PAK2
PAK3
PAK4
PAK6
PAK5
RHOA
CDC42
PPP3CA
PPP3CB
PPP3CC
CHP1
PPP3R1
PPP3R2
CHP2
NFAT5
NFATC1
NFATC2
NFATC3
NFATC4
SOS1
SOS2
HRAS
KRAS
NRAS
FOS
JUN
CARD11
BCL10
MALT1

CHUK
IKBKB
IKBKG
NFKB1
RELA
NFKBIA
NFKBIB
NFKBIE
CD28
ICOS
CD40LG
PIK3R5
PIK3R1
PIK3R2
PIK3R3
PIK3CA
PIK3CB
PIK3CD
PIK3CG
AKT3
AKT1
AKT2
MAP3K8
MAP3K14
PDCD1
CTLA4
PTPN6
CBLC
CBL
CBLB
IL2
IL4
IL5
IL10
IFNG
CSF2
TNF
CDK4
RASGRP1
PDK1
PLCG1
PRKCQ
TRAC
TRAJ1

TRAJ2
TRAJ3
TRAJ4
TRAJ5
TRAJ6
TRAJ7
TRAJ8
TRAJ9
TRAJ10
TRAJ11
TRAJ12
TRAJ13
TRAJ14
TRAJ15
TRAJ16
TRAJ17
TRAJ18
TRAJ19
TRAJ20
TRAJ21
TRAJ22
TRAJ23
TRAJ24
TRAJ25
TRAJ26
TRAJ27
TRAJ28
TRAJ29
TRAJ30
TRAJ31
TRAJ32
TRAJ33
TRAJ34
TRAJ35
TRAJ36
TRAJ37
TRAJ38
TRAJ39
TRAJ40
TRAJ41
TRAJ42
TRAJ43
TRAJ44
TRAJ45

TRAJ46
TRAJ47
TRAJ48
TRAJ49
TRAJ50
TRAJ52
TRAJ53
TRAJ54
TRAJ56
TRAJ57
TRAJ58
TRAJ59
TRAJ61
TRAV1-1
TRAV1-2
TRAV2
TRAV3
TRAV4
TRAV5
TRAV7
TRAV8-1
TRAV8-2
TRAV8-3
TRAV8-4
TRAV8-6
TRAV8-7
TRAV9-1
TRAV9-2
TRAV10
TRAV12-1
TRAV12-2
TRAV12-3
TRAV13-1
TRAV13-2
TRAV14DV4
TRAV16
TRAV17
TRAV18
TRAV19
TRAV20
TRAV21
TRAV22
TRAV23DV6
TRAV24

TRAV25
TRAV26-1
TRAV26-2
TRAV27
TRAV29DV5
TRAV30
TRAV34
TRAV35
TRAV36DV7
TRAV38-1
TRAV38-2DV8
TRAV39
TRAV40
TRAV41
TRBC1
TRBC2
TRBD1
TRBD2
TRBJ1-1
TRBJ1-2
TRBJ1-3
TRBJ1-4
TRBJ1-5
TRBJ1-6
TRBJ2-1
TRBJ2-2
TRBJ2-3
TRBJ2-4
TRBJ2-5
TRBJ2-6
TRBJ2-7
TRBV2
TRBV3-1
TRBV4-1
TRBV4-2
TRBV4-3
TRBV5-1
TRBV5-4
TRBV5-5
TRBV5-6
TRBV5-7
TRBV5-8
TRBV6-1
TRBV6-2

TRBV6-3
TRBV6-4
TRBV6-5
TRBV6-6
TRBV6-7
TRBV6-8
TRBV6-9
TRBV7-2
TRBV7-3
TRBV7-4
TRBV7-6
TRBV7-7
TRBV7-8
TRBV7-9
TRBV9
TRBV10-1
TRBV10-2
TRBV10-3
TRBV11-1
TRBV11-2
TRBV11-3
TRBV12-3
TRBV12-4
TRBV12-5
TRBV13
TRBV14
TRBV15
TRBV16
TRBV17
TRBV18
TRBV19
TRBV20-1
TRBV24-1
TRBV25-1
TRBV27
TRBV28
TRBV29-1
TRBV30
TRDC
TRDD1
TRDD2
TRDD3
TRDJ1
TRDJ2

TRDJ3
TRDJ4
TRDV1
TRDV2
TRDV3
TRGV9
TRGV8
TRGV5
TRGV4
TRGV3
TRGV2
TRGJP2
TRGJP1
TRGJP
TRGJ2
TRGJ1
TRGC2
TRGC1
TRAV6
BMP1
BMP10
BMP15
BMP2
BMP3
BMP4
BMP5
BMP6
BMP7
BMP8A
BMP8B
GDF1
GDF10
GDF11
GDF15
GDF2
GDF3
GDF5
GDF6
GDF7
GDF9
GDNF
INHA
INHBA
INHBB

INHBC
INHBE
LEFTY1
LEFTY2
NODAL
TGFB1
TGFB2
TGFB3
ACVR1B
ACVR1C
ACVR2A
ACVR2B
ACVRL1
AMHR2
BMPR1A
BMPR1B
BMPR2
TGFBR1
TGFBR2
TGFBR3
TNFRSF11B
TNFSF10
TNFSF11
TNFSF12
TNFSF13
TNFSF13B
TNFSF14
TNFSF15
TNFSF18
TNFSF4
TNFSF8
TNFSF9
TNFRSF10B
TNFRSF10C
TNFRSF10D
TNFRSF11A
TNFRSF12A
TNFRSF13B
TNFRSF13C
TNFRSF14
TNFRSF17
TNFRSF18
TNFRSF19
TNFRSF1A

TNFRSF1B
TNFRSF21
TNFRSF25
TNFRSF4
TNFRSF6B
TNFRSF8
TNFRSF9

Supplementary Table 2: The expression profiles of differentially expressed IRGs.

ID	CTSS	S100Z	STAT3	KLRK1	FPR1	C5AR2	RLN1	IFNGR2	IL2RB	IL17RA	IL6R
GSM3526790_Health	7.753835	7.562193	6.159127	6.523635	13.041375	4.177752	3.488679	7.429397	11.697615	10.516558	8.016731
GSM3526791_Health	8.05205	7.585128	6.332283	6.433114	13.059359	4.365086	3.98338	7.526709	11.184473	10.753404	8.099557
GSM3526792_Health	8.158303	7.615329	6.496448	7.032728	13.6144	4.812316	2.850973	7.841815	12.448046	10.938612	8.229915
GSM3526793_Health	8.606853	7.940073	6.892135	5.994068	13.90065	5.07392	2.817669	8.438085	11.176183	11.40461	8.954961
GSM3526794_Health	8.349323	8.019042	7.077913	6.208961	14.246335	5.231373	1.873653	8.180325	11.489816	11.387781	8.942967
GSM3526795_Health	8.479298	7.846417	7.133107	6.069453	13.948685	4.686719	2.765873	8.003079	11.532797	11.300405	8.531271
GSM3526796_Health	7.927365	7.752898	6.645509	6.778701	13.237102	4.396001	3.420402	7.73121	11.608161	10.830142	8.135098
GSM3526797_Health	8.358341	8.216398	6.774141	6.642801	12.937285	5.167076	2.425498	7.837108	11.755027	11.075362	8.422757
GSM4548691_Health	8.711288	7.806219	6.811867	6.443345	13.636464	5.325789	3.409073	7.896704	10.545664	10.718245	8.865022
GSM4548692_Health	9.072901	8.107681	7.015481	6.922884	14.553362	5.383786	2.925739	8.133585	10.833627	11.419146	9.025955
GSM4548693_Health	8.508565	8.154404	6.524103	7.259528	13.225172	4.406786	3.752731	7.700286	11.653686	11.100849	8.252157
GSM4548694_Health	8.112708	7.43185	6.870675	6.513924	14.147818	5.019726	2.673367	7.647896	11.259108	11.126068	8.706638
GSM4548695_Health	8.42766	7.426204	6.81426	5.91287	13.708865	5.541273	1.507592	8.004207	10.147838	10.767623	8.664221
GSM4548696_Health	8.482874	7.489565	7.148915	6.64505	13.473462	4.883556	4.493437	7.547515	11.320832	10.916284	8.531271
GSM4548697_Health	8.857537	8.496464	6.538614	6.38891	13.764036	5.260818	3.293304	8.064563	11.844722	11.243515	8.864427
GSM45486	8.731	8.0750	6.2941	6.46756	13.566	4.6479	3.299	7.67963	11.374	10.8531	8.446

98_Health	994	03	93	4	647	35	91	4	044	36	696
GSM45486	9.011	7.8619	6.5835	6.73287	13.948	5.0470	3.976	8.21769	11.493	11.1149	8.438
99_Health	968	22	92	8	685	33	097	3	239	71	085
GSM45487	8.514	8.1796	6.2762	6.33132	13.094	4.2246	4.309	7.70159	11.036	10.5443	7.993
00_Health	053	49	56	3	598	03	846	4	578	2	057
GSM45487	9.057	7.6559	7.1430	6.95617	13.219	5.4458	3.357	8.05418	11.456	11.1476	8.856
01_Health	198	41	14	5	76	63	759	1	523	5	162
GSM45487	8.521	8.4341	6.5663	7.24210	13.503	4.7578	3.400	7.57247	11.498	11.1103	8.515
02_Health	919	19	92	4	785	64	614		729	56	96
GSM45487	8.617	7.4467	7.0954	5.62521	14.161	5.8303	3.795	8.39581	10.139	10.8145	8.690
03_Health	172	08	67	8	291	17	556	5	956	61	707
GSM45487	8.726	7.8899	6.7791	7.13646	13.511	5.3250	3.269	8.99030	11.657	11.0478	8.253
04_Health	517	64	82	3	918	99	801	8	175	82	753
GSM45487	8.542	7.6094	6.6934	6.70408	12.973	5.2940	3.074	8.85558	11.208	10.9592	8.031
05_Health	435	2	35	4	289	2	556	7	456	91	192
GSM45487	8.477	7.6862	6.6368	6.34817	13.808	5.3313	3.339	8.73262	11.205	10.8879	8.085
06_Health	354	27	32	6	88	56	531	7	617	45	664
GSM45487	8.343	8.1371	6.9916	6.56737	13.888	5.4316	3.078	8.94785	11.659	11.0709	8.144
07_Health	157	29	2	2	561	56	765		893	52	105
GSM35267	8.714	8.6289	7.3828	5.12464	13.438	5.5787	1.447	8.55294	10.800	11.7518	9.500
82_IDD	941	74	28	3	306	26	806	2	203	42	53
GSM35267	8.388	8.4679	6.6907	5.89001	14.203	5.5503	2.916	8.13121	10.706	11.1908	9.071
83_IDD	909	03	42	3	52	37	284	7	173	59	36
GSM35267	9.287	7.6827	7.4318	6.18171	14.519	5.1944	2.576	8.37579	11.473	11.1844	8.617
84_IDD	805	11	5	6	419	75	475	5	142	73	172
GSM35267	8.151	8.3403	6.6607	6.84259	13.923	5.1562	2.456	8.25775	11.401	10.8286	8.022
85_IDD	613	43	98	8	922	25	955	4	449	11	404
GSM35267	8.792	8.4696	8.3097	5.64300	14.964	7.1022	1.639	8.99817	10.385	12.3882	10.46
86_IDD	046	76	99	5	209	24	793	6	952	23	0271
GSM35267	8.706	8.6939	6.9686	5.68312	13.847	5.1501	2.161	8.65655	10.655	11.3712	8.778
87_IDD	638	04	76	1	735	59	551	7	806	84	694
GSM35267	8.837	8.6698	6.8776	5.69794	14.232	5.3262	3.415	8.54499	10.813	11.5464	9.115
88_IDD	135	45	32	3	656	26	334	3	151	25	369
GSM35267	8.697	8.3682	7.1035	5.13626	14.029	5.3842	1.454	8.57886	9.7381	11.2158	8.839
89_IDD	476	64	34	2	542	18	214	3	3	5	647
GSM45487	8.549	7.9573	6.7176	6.84214	13.380	4.4879	5.635	7.92089	11.345	10.8131	8.552
08_IDD	704	64	2	8	741	36	868	2	174	51	942
GSM45487	8.864	8.2724	6.9720	6.83953	13.750	5.1976	3.916	8.29976	11.384	11.0731	8.790
09_IDD	427	51	97	6	878	31	21		865	04	72
GSM45487	8.926	8.8934	6.8757	7.30904	14.095	5.5960	2.896	8.05259	10.678	11.3028	9.088
10_IDD	635	59	97	7	227	77	033	4	711	11	74
GSM45487	8.889	7.9076	6.9883	5.92852	13.664	5.9261	2.536	8.06740	9.7615	11.0753	9.268
11_IDD	451	77	27	9	652	26	354	9	25	62	441
GSM45487	8.726	8.2749	6.8966	6.12799	13.833	5.1574	1.444	8.10937	10.643	11.1311	8.714

12_IDD	517	24	89		947	15	85	1	811	2	254
GSM4548713_IDD	9.008775	8.282314	7.492494	5.502062	13.48005	5.903606	1.408131	8.770649	10.442067	11.578468	9.383306
GSM4548714_IDD	9.069791	7.740152	6.977982	5.742279	14.039345	5.026502	2.550795	8.092934	11.392834	11.363734	8.918958
GSM4548715_IDD	9.292053	8.717961	7.739503	5.386356	14.843535	5.715678	1.414954	8.70089	10.515029	11.659893	9.438429
GSM4548716_IDD	8.75634	9.259339	7.078326	5.428223	14.081112	5.922311	2.327994	8.258286	10.792271	11.41033	9.202372
GSM4548717_IDD	9.061141	8.686223	6.279968	5.318216	13.664652	5.653789	1.58423	8.458894	10.258311	10.490204	8.208946
GSM4548718_IDD	8.837135	8.771921	6.659491	5.942086	14.016953	5.149693	3.655789	8.306592	11.292996	11.245834	8.719313
GSM4548719_IDD	8.620941	8.277881	6.927038	5.917384	13.90065	5.313456	2.611452	8.227079	10.354452	11.015279	8.763221
GSM4548720_IDD	8.936157	7.366467	7.484895	6.256527	14.729161	5.900396	1.48234	9.735978	11.345174	11.320832	8.423402
GSM4548721_IDD	8.556679	7.749719	7.114233	6.323474	14.029542	5.420863	3.175367	9.247964	11.122104	11.66629	8.636518
GSM4548722_IDD	9.027443	8.014086	7.812574	6.168264	14.26401	6.3022	1.37956	9.614078	10.548361	11.595987	8.793258
GSM4548723_IDD	9.010479	8.113908	7.581306	5.87581	15.075947	6.337263	1.382715	9.620975	10.660828	11.99733	8.937859
GSM4548724_IDD	8.812452	8.40434	7.354223	6.025335	14.519419	5.538225	3.039735	9.310843	10.707739	11.546425	8.668682

Supplementary Table 3: GO enrichment analysis of DEGs.

ONTOLOGY	ID	Description	pvalue	Count
BP	GO:0019221	cytokine-mediated signaling pathway	4.39E-06	17
BP	GO:0048872	homeostasis of number of cells	8.19E-05	11
BP	GO:0002181	cytoplasmic translation	0.00010917	8
BP	GO:0071709	membrane assembly	0.000128534	5
BP	GO:0001765	membrane raft assembly	0.000145385	3
BP	GO:0044091	membrane biogenesis	0.000202836	5
BP	GO:0032640	tumor necrosis factor production	0.000430461	8
BP	GO:0032680	regulation of tumor necrosis factor production	0.000430461	8

BP	GO:0050727	regulation of inflammatory response	0.000450808	12
BP	GO:0032677	regulation of interleukin-8 production	0.000512154	6
BP	GO:0071706	tumor necrosis factor superfamily cytokine production	0.000515788	8
BP	GO:1903555	regulation of tumor necrosis factor superfamily cytokine production	0.000515788	8
BP	GO:0032637	interleukin-8 production	0.000539459	6
BP	GO:0032496	response to lipopolysaccharide	0.000601834	11
BP	GO:0071800	podosome assembly	0.000683457	3
BP	GO:1902622	regulation of neutrophil migration	0.000688155	4
BP	GO:0046782	regulation of viral transcription	0.000805759	3
BP	GO:0002526	acute inflammatory response	0.000839402	6
BP	GO:0046632	alpha-beta T cell differentiation	0.000839402	6
BP	GO:0002262	myeloid cell homeostasis	0.000933209	7
BP	GO:0016553	base conversion or substitution editing	0.000941127	3
BP	GO:0002237	response to molecule of bacterial origin	0.000956923	11
BP	GO:0032635	interleukin-6 production	0.0012461	7
BP	GO:0032675	regulation of interleukin-6 production	0.0012461	7
BP	GO:0002224	toll-like receptor signaling pathway	0.001254154	6
BP	GO:0043367	CD4-positive, alpha-beta T cell differentiation	0.001356946	5
BP	GO:0002758	innate immune response-activating signal transduction	0.001430728	3
BP	GO:0060353	regulation of cell adhesion molecule production	0.001430728	3
BP	GO:0019083	viral transcription	0.001461088	4
BP	GO:0007259	receptor signaling pathway via JAK-STAT	0.001479908	7
BP	GO:0002221	pattern recognition receptor signaling pathway	0.001582479	7
BP	GO:0034101	erythrocyte homeostasis	0.001740462	6
BP	GO:0031579	membrane raft organization	0.001831486	3
BP	GO:0060352	cell adhesion molecule production	0.002055512	3
BP	GO:0097696	receptor signaling pathway via	0.002114431	7

		STAT		
BP	GO:0060333	interferon-gamma-mediated signaling pathway	0.002295857	3
BP	GO:0001819	positive regulation of cytokine production	0.002303058	12
BP	GO:0002764	immune response-regulating signaling pathway	0.002343788	12
BP	GO:0019080	viral gene expression	0.002352722	5
BP	GO:0051258	protein polymerization	0.002748019	9
BP	GO:0032731	positive regulation of interleukin-1 beta production	0.003230446	4
BP	GO:0032757	positive regulation of interleukin-8 production	0.003230446	4
BP	GO:1903131	mononuclear cell differentiation	0.003350073	11
BP	GO:0035710	CD4-positive, alpha-beta T cell activation	0.003352731	5
BP	GO:0046425	regulation of receptor signaling pathway via JAK-STAT	0.003496265	5
BP	GO:0070542	response to fatty acid	0.003624005	4
BP	GO:0050900	leukocyte migration	0.003626051	10
BP	GO:0042093	T-helper cell differentiation	0.004048908	4
BP	GO:0050901	leukocyte tethering or rolling	0.004101648	3
BP	GO:0071763	nuclear membrane organization	0.004101648	3
BP	GO:0010728	regulation of hydrogen peroxide biosynthetic process	0.004104161	2
BP	GO:0046631	alpha-beta T cell activation	0.00447497	6
BP	GO:0002294	CD4-positive, alpha-beta T cell differentiation involved in immune response	0.004506286	4
BP	GO:0030099	myeloid cell differentiation	0.00453515	10
BP	GO:1902600	proton transmembrane transport	0.004615702	6
BP	GO:0071222	cellular response to lipopolysaccharide	0.004681427	7
BP	GO:0002287	alpha-beta T cell activation involved in immune response	0.004747499	4
BP	GO:0002293	alpha-beta T cell differentiation involved in immune response	0.004747499	4
BP	GO:1903238	positive regulation of leukocyte tethering or rolling	0.004983889	2
BP	GO:0045123	cellular extravasation	0.004997243	4
BP	GO:0002691	regulation of cellular extravasation	0.005253332	3
BP	GO:1901658	glycosyl compound catabolic process	0.005253332	3

BP	GO:0022904	respiratory electron transport chain	0.005379226	5
BP	GO:1904892	regulation of receptor signaling pathway via STAT	0.005379226	5
BP	GO:0048638	regulation of developmental growth	0.005461216	9
BP	GO:0050792	regulation of viral process	0.005693873	6
BP	GO:0032732	positive regulation of interleukin-1 production	0.005798989	4
BP	GO:0008154	actin polymerization or depolymerization	0.00587017	7
BP	GO:0002366	leukocyte activation involved in immune response	0.005941275	8
BP	GO:0043270	positive regulation of ion transport	0.005941275	8
BP	GO:0016554	cytidine to uridine editing	0.005942178	2
BP	GO:0030259	lipid glycosylation	0.005942178	2
BP	GO:0071398	cellular response to fatty acid	0.006118559	3
BP	GO:0071219	cellular response to molecule of bacterial origin	0.00631263	7
BP	GO:0002292	T cell differentiation involved in immune response	0.006378544	4
BP	GO:0034121	regulation of toll-like receptor signaling pathway	0.006378544	4
BP	GO:0002263	cell activation involved in immune response	0.006463104	8
BP	GO:0030218	erythrocyte differentiation	0.006663372	5
BP	GO:0002430	complement receptor mediated signaling pathway	0.006977415	2
BP	GO:0006000	fructose metabolic process	0.006977415	2
BP	GO:0021554	optic nerve development	0.006977415	2
BP	GO:1990266	neutrophil migration	0.007135335	5
BP	GO:1901136	carbohydrate derivative catabolic process	0.007139405	6
BP	GO:0032733	positive regulation of interleukin-10 production	0.007567369	3
BP	GO:0007100	mitotic centrosome separation	0.008088012	2
BP	GO:0031468	nuclear membrane reassembly	0.008088012	2
BP	GO:0046131	pyrimidine ribonucleoside metabolic process	0.008088012	2
BP	GO:0046133	pyrimidine ribonucleoside catabolic process	0.008088012	2
BP	GO:0071260	cellular response to mechanical stimulus	0.00834438	4

BP	GO:0042088	T-helper 1 type immune response	0.008636327	3
BP	GO:0032102	negative regulation of response to external stimulus	0.008779902	10
BP	GO:0046427	positive regulation of receptor signaling pathway via JAK-STAT	0.009202285	3
BP	GO:0032897	negative regulation of viral transcription	0.009272405	2
BP	GO:0045064	T-helper 2 cell differentiation	0.009272405	2
BP	GO:0050746	regulation of lipoprotein metabolic process	0.009272405	2
BP	GO:0051299	centrosome separation	0.009272405	2
BP	GO:0055001	muscle cell development	0.009781038	6
BP	GO:0048546	digestive tract morphogenesis	0.009789448	3
BP	GO:0034138	toll-like receptor 3 signaling pathway	0.010529053	2
BP	GO:0046135	pyrimidine nucleoside catabolic process	0.010529053	2
BP	GO:0048557	embryonic digestive tract morphogenesis	0.010529053	2
BP	GO:1903236	regulation of leukocyte tethering or rolling	0.010529053	2
BP	GO:0140694	non-membrane-bounded organelle assembly	0.010583393	9
BP	GO:0019646	aerobic electron transport chain	0.010670311	4
BP	GO:0019915	lipid storage	0.010670311	4
BP	GO:0071216	cellular response to biotic stimulus	0.011025035	7
BP	GO:0006998	nuclear envelope organization	0.011027979	3
BP	GO:0009261	ribonucleotide catabolic process	0.011027979	3
BP	GO:0008203	cholesterol metabolic process	0.011440815	5
BP	GO:0030041	actin filament polymerization	0.011609579	6
BP	GO:0002673	regulation of acute inflammatory response	0.011679616	3
BP	GO:2000811	negative regulation of anoikis	0.011856435	2
BP	GO:0006953	acute-phase response	0.012352994	3
BP	GO:1904894	positive regulation of receptor signaling pathway via STAT	0.012352994	3
BP	GO:1990573	potassium ion import across plasma membrane	0.012352994	3
BP	GO:0055006	cardiac cell development	0.012432233	4
BP	GO:0002283	neutrophil activation involved in immune response	0.013253058	2

BP	GO:0034162	toll-like receptor 9 signaling pathway	0.013253058	2
BP	GO:0048643	positive regulation of skeletal muscle tissue development	0.013253058	2
BP	GO:0050665	hydrogen peroxide biosynthetic process	0.013253058	2
BP	GO:0110154	RNA decapping	0.013253058	2
BP	GO:0032755	positive regulation of interleukin-6 production	0.013378877	4
BP	GO:0030217	T cell differentiation	0.01375336	7
BP	GO:0042773	ATP synthesis coupled electron transport	0.014370244	4
BP	GO:0042775	mitochondrial ATP synthesis coupled electron transport	0.014370244	4
BP	GO:0002218	activation of innate immune response	0.014504611	3
BP	GO:0002523	leukocyte migration involved in inflammatory response	0.014717446	2
BP	GO:0034620	cellular response to unfolded protein	0.014882905	4
BP	GO:0001889	liver development	0.015132674	5
BP	GO:1902652	secondary alcohol metabolic process	0.015132674	5
BP	GO:0097530	granulocyte migration	0.015540797	5
BP	GO:0045063	T-helper 1 cell differentiation	0.016248148	2
BP	GO:0045844	positive regulation of striated muscle tissue development	0.016248148	2
BP	GO:0048636	positive regulation of muscle organ development	0.016248148	2
BP	GO:0097320	plasma membrane tubulation	0.016248148	2
BP	GO:0061008	hepaticobiliary system development	0.016378977	5
BP	GO:0016125	sterol metabolic process	0.017246743	5
BP	GO:0051092	positive regulation of NF-kappaB transcription factor activity	0.017246743	5
BP	GO:0042454	ribonucleoside catabolic process	0.017843734	2
BP	GO:0050908	detection of light stimulus involved in visual perception	0.017843734	2
BP	GO:0050962	detection of light stimulus involved in sensory perception	0.017843734	2
BP	GO:1901863	positive regulation of muscle tissue development	0.017843734	2
BP	GO:2000047	regulation of cell-cell adhesion	0.017843734	2

		mediated by cadherin		
BP	GO:0051962	positive regulation of nervous system development	0.018212045	7
BP	GO:0061756	leukocyte adhesion to vascular endothelial cell	0.018533677	3
BP	GO:0032760	positive regulation of tumor necrosis factor production	0.018795496	4
BP	GO:0032481	positive regulation of type I interferon production	0.019406457	3
BP	GO:2000736	regulation of stem cell differentiation	0.019406457	3
BP	GO:0002220	innate immune response activating cell surface receptor signaling pathway	0.019502795	2
BP	GO:0009220	pyrimidine ribonucleotide biosynthetic process	0.019502795	2
BP	GO:0010310	regulation of hydrogen peroxide metabolic process	0.019502795	2
BP	GO:0062207	regulation of pattern recognition receptor signaling pathway	0.020019571	4
BP	GO:0031349	positive regulation of defense response	0.020251855	7
BP	GO:0046620	regulation of organ growth	0.020649619	4
BP	GO:0031663	lipopolysaccharide-mediated signaling pathway	0.021219251	3
BP	GO:0032740	positive regulation of interleukin-17 production	0.021223945	2
BP	GO:2000737	negative regulation of stem cell differentiation	0.021223945	2
BP	GO:1903557	positive regulation of tumor necrosis factor superfamily cytokine production	0.021291749	4
BP	GO:0034764	positive regulation of transmembrane transport	0.021345928	6
BP	GO:0016032	viral process	0.021797853	9
BP	GO:1905515	non-motile cilium assembly	0.022159295	3
BP	GO:0042110	T cell activation	0.022454264	10
BP	GO:0050657	nucleic acid transport	0.022565287	5
BP	GO:0050658	RNA transport	0.022565287	5
BP	GO:0061025	membrane fusion	0.022565287	5
BP	GO:0002755	MyD88-dependent toll-like receptor signaling pathway	0.023005815	2
BP	GO:0006213	pyrimidine nucleoside metabolic process	0.023005815	2

BP	GO:0010155	regulation of proton transport	0.023005815	2
BP	GO:0036003	positive regulation of transcription from RNA polymerase II promoter in response to stress	0.023005815	2
BP	GO:0048641	regulation of skeletal muscle tissue development	0.023005815	2
BP	GO:0070935	3'-UTR-mediated mRNA stabilization	0.023005815	2
BP	GO:2000209	regulation of anoikis	0.023005815	2
BP	GO:0002274	myeloid leukocyte activation	0.023083124	6
BP	GO:0032613	interleukin-10 production	0.023121779	3
BP	GO:0032653	regulation of interleukin-10 production	0.023121779	3
BP	GO:0032611	interleukin-1 beta production	0.02329115	4
BP	GO:0032651	regulation of interleukin-1 beta production	0.02329115	4
BP	GO:0050769	positive regulation of neurogenesis	0.023986399	6
BP	GO:0097193	intrinsic apoptotic signaling pathway	0.023997594	7
BP	GO:1902373	negative regulation of mRNA catabolic process	0.024106699	3
BP	GO:0051236	establishment of RNA localization	0.024181024	5
BP	GO:0034767	positive regulation of ion transmembrane transport	0.024735741	5
BP	GO:0002460	adaptive immune response based on somatic recombination of immune receptors built from immunoglobulin superfamily domains	0.024758442	8
BP	GO:0035336	long-chain fatty-acyl-CoA metabolic process	0.024847061	2
BP	GO:0042832	defense response to protozoan	0.024847061	2
BP	GO:0046835	carbohydrate phosphorylation	0.024847061	2
BP	GO:1904996	positive regulation of leukocyte adhesion to vascular endothelial cell	0.024847061	2
BP	GO:0098657	import into cell	0.024913103	6
BP	GO:0046530	photoreceptor cell differentiation	0.025114044	3
BP	GO:0002429	immune response-activating cell surface receptor signaling	0.025208543	7

		pathway		
BP	GO:0002757	immune response-activating signal transduction	0.025208543	7
BP	GO:0032103	positive regulation of response to external stimulus	0.025593771	9
BP	GO:0002286	T cell activation involved in immune response	0.026129137	4
BP	GO:0048524	positive regulation of viral process	0.026143798	3
BP	GO:0061512	protein localization to cilium	0.026143798	3
BP	GO:0045333	cellular respiration	0.026347578	6
BP	GO:0001562	response to protozoan	0.026746357	2
BP	GO:0009164	nucleoside catabolic process	0.026746357	2
BP	GO:1900017	positive regulation of cytokine production involved in inflammatory response	0.026746357	2
BP	GO:0046626	regulation of insulin receptor signaling pathway	0.027195936	3
BP	GO:0050922	negative regulation of chemotaxis	0.027195936	3
BP	GO:1902476	chloride transmembrane transport	0.027195936	3
BP	GO:0008286	insulin receptor signaling pathway	0.02762264	4
BP	GO:0035967	cellular response to topologically incorrect protein	0.02762264	4
BP	GO:0048736	appendage development	0.027631977	5
BP	GO:0060173	limb development	0.027631977	5
BP	GO:1903828	negative regulation of cellular protein localization	0.028388145	4
BP	GO:0032703	negative regulation of interleukin-2 production	0.028702397	2
BP	GO:0001659	temperature homeostasis	0.028848332	5
BP	GO:0048639	positive regulation of developmental growth	0.028848332	5
BP	GO:0022900	electron transport chain	0.029469035	5
BP	GO:0019318	hexose metabolic process	0.029905697	6
BP	GO:0050728	negative regulation of inflammatory response	0.030098127	5
BP	GO:0051090	regulation of DNA-binding transcription factor activity	0.030212471	9
BP	GO:0045936	negative regulation of phosphate metabolic process	0.03059039	9
BP	GO:0002675	positive regulation of acute	0.030713896	2

		inflammatory response		
BP	GO:0009312	oligosaccharide biosynthetic process	0.030713896	2
BP	GO:0034123	positive regulation of toll-like receptor signaling pathway	0.030713896	2
BP	GO:0044331	cell-cell adhesion mediated by cadherin	0.030713896	2
BP	GO:0046426	negative regulation of receptor signaling pathway via JAK-STAT	0.030713896	2
BP	GO:0098739	import across plasma membrane	0.030735636	5
BP	GO:0010563	negative regulation of phosphorus metabolic process	0.030971608	9
BP	GO:0060415	muscle tissue morphogenesis	0.031627613	3
BP	GO:0030098	lymphocyte differentiation	0.031866367	8
BP	GO:0018108	peptidyl-tyrosine phosphorylation	0.032298223	8
BP	GO:0003209	cardiac atrium morphogenesis	0.032779586	2
BP	GO:0009218	pyrimidine ribonucleotide metabolic process	0.032779586	2
BP	GO:0010458	exit from mitosis	0.032779586	2
BP	GO:0048679	regulation of axon regeneration	0.032779586	2
BP	GO:1902175	regulation of oxidative stress-induced intrinsic apoptotic signaling pathway	0.032779586	2
BP	GO:0022407	regulation of cell-cell adhesion	0.033328862	9
BP	GO:0018212	peptidyl-tyrosine modification	0.033617809	8
BP	GO:0098656	anion transmembrane transport	0.034342631	6
BP	GO:0002819	regulation of adaptive immune response	0.034738927	5
BP	GO:0000132	establishment of mitotic spindle orientation	0.034898222	2
BP	GO:0006221	pyrimidine nucleotide biosynthetic process	0.034898222	2
BP	GO:0009264	deoxyribonucleotide catabolic process	0.034898222	2
BP	GO:0070498	interleukin-1-mediated signaling pathway	0.034898222	2
BP	GO:2000637	positive regulation of gene silencing by miRNA	0.034898222	2
BP	GO:0009166	nucleotide catabolic process	0.035184272	3
BP	GO:0032535	regulation of cellular component size	0.035898027	8
BP	GO:0042692	muscle cell differentiation	0.036366329	8

BP	GO:0002437	inflammatory response to antigenic stimulus	0.036413853	3
BP	GO:0030968	endoplasmic reticulum unfolded protein response	0.036413853	3
BP	GO:0002768	immune response-regulating cell surface receptor signaling pathway	0.036429139	7
BP	GO:0042326	negative regulation of phosphorylation	0.03683875	8
BP	GO:0043123	positive regulation of I-kappaB kinase/NF-kappaB signaling	0.036856225	5
BP	GO:0034656	nucleobase-containing small molecule catabolic process	0.037068576	2
BP	GO:0045066	regulatory T cell differentiation	0.037068576	2
BP	GO:0046386	deoxyribose phosphate catabolic process	0.037068576	2
BP	GO:0048566	embryonic digestive tract development	0.037068576	2
BP	GO:0055075	potassium ion homeostasis	0.037068576	2
BP	GO:0060148	positive regulation of posttranscriptional gene silencing	0.037068576	2
BP	GO:0032612	interleukin-1 production	0.037641912	4
BP	GO:0032652	regulation of interleukin-1 production	0.037641912	4
BP	GO:1902369	negative regulation of RNA catabolic process	0.037665324	3
BP	GO:0031100	animal organ regeneration	0.038938603	3
BP	GO:0032720	negative regulation of tumor necrosis factor production	0.038938603	3
BP	GO:0071496	cellular response to external stimulus	0.039124055	7
BP	GO:0060828	regulation of canonical Wnt signaling pathway	0.039185318	6
BP	GO:0090022	regulation of neutrophil chemotaxis	0.039289438	2
BP	GO:1901532	regulation of hematopoietic progenitor cell differentiation	0.039289438	2
BP	GO:0007098	centrosome cycle	0.039489609	4
BP	GO:0051028	mRNA transport	0.039489609	4
BP	GO:0046390	ribose phosphate biosynthetic process	0.039800475	5
BP	GO:0048644	muscle organ morphogenesis	0.040233601	3
BP	GO:0045089	positive regulation of innate	0.040432589	4

		immune response		
BP	GO:1903556	negative regulation of tumor necrosis factor superfamily cytokine production	0.041550226	3
BP	GO:0070570	regulation of neuron projection regeneration	0.041559618	2
BP	GO:0071539	protein localization to centrosome	0.041559618	2
BP	GO:0061564	axon development	0.041608634	9
BP	GO:0005996	monosaccharide metabolic process	0.041761759	6
BP	GO:0019693	ribose phosphate metabolic process	0.042310716	8
BP	GO:0006997	nucleus organization	0.042356821	4
BP	GO:0046328	regulation of JNK cascade	0.042356821	4
BP	GO:0002285	lymphocyte activation involved in immune response	0.042884212	5
BP	GO:0050772	positive regulation of axonogenesis	0.042888381	3
BP	GO:0051051	negative regulation of transport	0.043032576	9
BP	GO:0030111	regulation of Wnt signaling pathway	0.043702347	7
BP	GO:0051091	positive regulation of DNA-binding transcription factor activity	0.043762767	6
BP	GO:0010614	negative regulation of cardiac muscle hypertrophy	0.043877944	2
BP	GO:0043276	anoikis	0.043877944	2
BP	GO:0055094	response to lipoprotein particle	0.043877944	2
BP	GO:1904893	negative regulation of receptor signaling pathway via STAT	0.043877944	2
BP	GO:0042157	lipoprotein metabolic process	0.044332075	4
BP	GO:0031667	response to nutrient levels	0.044981841	9
BP	GO:0031330	negative regulation of cellular catabolic process	0.045129681	6
BP	GO:0031099	regeneration	0.046108292	5
BP	GO:0050864	regulation of B cell activation	0.046108292	5
BP	GO:0006739	NADP metabolic process	0.046243261	2
BP	GO:0007020	microtubule nucleation	0.046243261	2
BP	GO:0009119	ribonucleoside metabolic process	0.046243261	2
BP	GO:0040001	establishment of mitotic spindle localization	0.046243261	2
BP	GO:0072528	pyrimidine-containing	0.046243261	2

		compound biosynthetic process		
BP	GO:0097421	liver regeneration	0.046243261	2
BP	GO:1905508	protein localization to microtubule organizing center	0.046243261	2
BP	GO:0006986	response to unfolded protein	0.046358314	4
BP	GO:0072527	pyrimidine-containing compound metabolic process	0.047030993	3
BP	GO:0034655	nucleobase-containing compound catabolic process	0.048298123	8
BP	GO:1901292	nucleoside phosphate catabolic process	0.048454211	3
BP	GO:0006403	RNA localization	0.048618842	5
BP	GO:0003230	cardiac atrium development	0.048654433	2
BP	GO:0014741	negative regulation of muscle hypertrophy	0.048654433	2
BP	GO:0042092	type 2 immune response	0.048654433	2
BP	GO:0042119	neutrophil activation	0.048654433	2
BP	GO:0042755	eating behavior	0.048654433	2
BP	GO:0071402	cellular response to lipoprotein particle stimulus	0.048654433	2
BP	GO:0060271	cilium assembly	0.049252007	7
BP	GO:0051348	negative regulation of transferase activity	0.049389424	6
BP	GO:0032370	positive regulation of lipid transport	0.049898409	3
CC	GO:0030667	secretory granule membrane	4.36E-07	15
CC	GO:0001931	uropod	5.89E-06	4
CC	GO:0031254	cell trailing edge	5.89E-06	4
CC	GO:0022626	cytosolic ribosome	7.18E-06	8
CC	GO:0019898	extrinsic component of membrane	5.47E-05	12
CC	GO:0044391	ribosomal subunit	7.14E-05	9
CC	GO:0005840	ribosome	0.000130025	10
CC	GO:1904813	ficolin-1-rich granule lumen	0.000218783	7
CC	GO:0042788	polysomal ribosome	0.000256273	4
CC	GO:0005774	vacuolar membrane	0.000441408	13
CC	GO:0101002	ficolin-1-rich granule	0.000480615	8
CC	GO:0005765	lysosomal membrane	0.000528693	12
CC	GO:0098852	lytic vacuole membrane	0.000528693	12
CC	GO:0022627	cytosolic small ribosomal subunit	0.000739224	4
CC	GO:0005925	focal adhesion	0.000866979	12
CC	GO:0030055	cell-substrate junction	0.001000218	12
CC	GO:0042581	specific granule	0.001010252	7

CC	GO:0030864	cortical actin cytoskeleton	0.001060687	5
CC	GO:0035579	specific granule membrane	0.001992272	5
CC	GO:0032580	Golgi cisterna membrane	0.002405758	5
CC	GO:0022625	cytosolic large ribosomal subunit	0.002481971	4
CC	GO:0035577	azurophil granule membrane	0.002481971	4
CC	GO:0005844	polysome	0.003969018	4
CC	GO:0034451	centriolar satellite	0.004019384	5
CC	GO:0030863	cortical cytoskeleton	0.004181781	5
CC	GO:0005766	primary lysosome	0.004223693	6
CC	GO:0005795	Golgi stack	0.004223693	6
CC	GO:0042582	azurophil granule	0.004223693	6
CC	GO:0015934	large ribosomal subunit	0.005064683	5
CC	GO:0015935	small ribosomal subunit	0.005152942	4
CC	GO:0044853	plasma membrane raft	0.005653709	5
CC	GO:0031985	Golgi cisterna	0.006740289	5
CC	GO:0070069	cytochrome complex	0.007966363	3
CC	GO:0005901	caveola	0.009280595	4
CC	GO:0000153	cytoplasmic ubiquitin ligase complex	0.010413073	2
CC	GO:0005746	mitochondrial respirasome	0.0113121	4
CC	GO:0098803	respiratory chain complex	0.011749984	4
CC	GO:0098562	cytoplasmic side of membrane	0.013029482	6
CC	GO:0034774	secretory granule lumen	0.014080014	8
CC	GO:0060205	cytoplasmic vesicle lumen	0.014811351	8
CC	GO:0031983	vesicle lumen	0.015314033	8
CC	GO:0005769	early endosome	0.015652976	9
CC	GO:0005751	mitochondrial respiratory chain complex IV	0.016071418	2
CC	GO:0070469	respirasome	0.016733898	4
CC	GO:0031234	extrinsic component of cytoplasmic side of plasma membrane	0.017294542	4
CC	GO:0045121	membrane raft	0.017449207	8
CC	GO:0098857	membrane microdomain	0.017449207	8
CC	GO:0030139	endocytic vesicle	0.017730428	8
CC	GO:0042470	melanosome	0.022202711	4
CC	GO:0048770	pigment granule	0.022202711	4
CC	GO:0031225	anchored component of membrane	0.025898117	5
CC	GO:0033176	proton-transporting V-type ATPase complex	0.026460951	2
CC	GO:0009898	cytoplasmic side of plasma membrane	0.027058697	5

CC	GO:0001891	phagocytic cup	0.03038825	2
CC	GO:0005942	phosphatidylinositol 3-kinase complex	0.03038825	2
CC	GO:0045277	respiratory chain complex IV	0.03038825	2
CC	GO:0048787	presynaptic active zone membrane	0.03038825	2
CC	GO:0005938	cell cortex	0.032036804	7
CC	GO:0002102	podosome	0.032433156	2
CC	GO:0044291	cell-cell contact zone	0.033488796	3
CC	GO:0048786	presynaptic active zone	0.03468034	3
CC	GO:0009925	basal plasma membrane	0.040825077	6
CC	GO:0005791	rough endoplasmic reticulum	0.046372594	3
MF	GO:0140375	immune receptor activity	1.62E-05	9
MF	GO:0003735	structural constituent of ribosome	1.69E-05	10
MF	GO:0022884	macromolecule transmembrane transporter activity	0.002498877	3
MF	GO:0004896	cytokine receptor activity	0.003066703	5
MF	GO:0019203	carbohydrate phosphatase activity	0.004350905	2
MF	GO:0050308	sugar-phosphatase activity	0.004350905	2
MF	GO:0004875	complement receptor activity	0.006296949	2
MF	GO:0032396	inhibitory MHC class I receptor activity	0.006296949	2
MF	GO:0017110	nucleoside-diphosphatase activity	0.008567543	2
MF	GO:0035259	glucocorticoid receptor binding	0.008567543	2
MF	GO:0046935	1-phosphatidylinositol-3-kinase regulator activity	0.011148958	2
MF	GO:0032393	MHC class I receptor activity	0.012552048	2
MF	GO:0042625	ATPase-coupled ion transmembrane transporter activity	0.012552048	2
MF	GO:0044769	ATPase activity, coupled to transmembrane movement of ions, rotational mechanism	0.012552048	2
MF	GO:0046961	proton-transporting ATPase activity, rotational mechanism	0.012552048	2
MF	GO:0030228	lipoprotein particle receptor activity	0.014027884	2
MF	GO:0009678	pyrophosphate hydrolysis-driven proton transmembrane transporter activity	0.017191413	2
MF	GO:0035014	phosphatidylinositol 3-kinase	0.017191413	2

		regulator activity		
MF	GO:0005123	death receptor binding	0.018875979	2
MF	GO:0019200	carbohydrate kinase activity	0.018875979	2
MF	GO:0008320	protein transmembrane transporter activity	0.020627034	2
MF	GO:0033691	sialic acid binding	0.020627034	2
MF	GO:0019865	immunoglobulin binding	0.022443075	2
MF	GO:0022804	active transmembrane transporter activity	0.022809004	9
MF	GO:0051015	actin filament binding	0.023339782	6
MF	GO:0019957	C-C chemokine binding	0.024322619	2
MF	GO:0005242	inward rectifier potassium channel activity	0.026264211	2
MF	GO:0035255	ionotropic glutamate receptor binding	0.030327818	2
MF	GO:0140326	ATPase-coupled intramembrane lipid transporter activity	0.030327818	2
MF	GO:0003953	NAD+ nucleosidase activity	0.03244703	2
MF	GO:0015399	primary active transmembrane transporter activity	0.037203689	5
MF	GO:0031210	phosphatidylcholine binding	0.039137941	2
MF	GO:0140318	protein transporter activity	0.039137941	2
MF	GO:0030332	cyclin binding	0.041474917	2
MF	GO:0015078	proton transmembrane transporter activity	0.043272358	4
MF	GO:0038024	cargo receptor activity	0.043328165	3
MF	GO:0019956	chemokine binding	0.043863072	2
MF	GO:0002020	protease binding	0.048526249	4

Supplementary Table 4: The DEGs.

id	logFC	AveExpr	t	P.Value	adj.P.Val	B
PSTPIP2	1.17525155	5.83726336	6.550797576	6.94E-07	0.018027149	5.790897949
FAM160B1	0.7239032	7.35618524	6.066007246	2.34E-06	0.020411103	4.723876964
LOC101928737	0.74818395	1.48858284	5.888611119	3.67E-06	0.020411103	4.325816575
PACSIN2	1.02620925	7.704487	5.838292417	4.17E-06	0.020411103	4.212228341
FCGR2C	1.0578817	12.18741544	5.82717075	4.29E-06	0.020411103	4.187083627
B3GNTL1	1.3012871	5.62051692	5.720672404	5.63E-06	0.020411103	3.945610823
STX3	1.072545	6.9556232	5.713937364	5.73E-06	0.020411103	3.930298641
RPL30	-1.01068325	14.3990862	-5.677479551	6.29E-06	0.020411103	3.847328687
GUSBP10	1.2135186	1.81681532	5.600469932	7.66E-06	0.021852621	3.671623967
CHMP2A	0.59356	11.7160654	5.563991067	8.42E-06	0.021852621	3.588188718
MSRB1	1.0009062	12.22285644	5.487412418	1.03E-05	0.024195155	3.412626085

BCL3	1.2087617	11.15118864	5.388147794	1.32E-05	0.025372866	3.184271856
HIATL1	0.75667965	7.58382588	5.344808607	1.48E-05	0.025372866	3.084312054
KCNJ2	0.92102255	10.77260256	5.329608589	1.54E-05	0.025372866	3.049218379
STAT3	0.81309815	7.13513688	5.298172575	1.67E-05	0.025372866	2.9765824
FPR1	0.842759	14.1011652	5.293583232	1.69E-05	0.025372866	2.96597199
ECE1	1.36907735	7.00671712	5.278388583	1.76E-05	0.025372866	2.930831251
ACSL1	1.29744025	10.8978666	5.21757034	2.06E-05	0.025405436	2.790009487
ZDHHC18	1.1825623	8.03259896	5.213040429	2.08E-05	0.025405436	2.779510337
OR1M1	0.70105875	1.6692126	5.211531203	2.09E-05	0.025405436	2.776012035
PIK3R5	0.67634765	5.94784208	5.196310751	2.18E-05	0.025405436	2.740723298
GABRR2	1.03465425	1.5452684	5.131411665	2.58E-05	0.025405436	2.590085468
NADK	1.0555282	8.33730384	5.13112455	2.58E-05	0.025405436	2.589418459
PFKFB4	0.9794875	9.0387138	5.122608302	2.63E-05	0.025405436	2.569631725
SLA	0.86563005	12.77647296	5.119758967	2.65E-05	0.025405436	2.563010585
GBAP1	0.96173015	6.02302008	5.086379024	2.89E-05	0.025405436	2.485408634
TRIM27	0.8035533	8.51747036	5.076837037	2.97E-05	0.025405436	2.463213684
KCNJ15	1.19734785	8.24043392	5.071633666	3.01E-05	0.025405436	2.451108362
TMEM180	0.94558615	3.95903368	5.055173218	3.14E-05	0.025405436	2.412804437
BIN2	0.9031949	9.54555748	5.034272288	3.31E-05	0.025405436	2.36414669
FRAT1	0.95594125	7.574162	5.032824291	3.33E-05	0.025405436	2.360774891
MCL1	1.03778225	10.3908276	5.015279599	3.48E-05	0.02583157	2.319911984
HN1	0.95176585	10.93731832	4.995330757	3.67E-05	0.026451468	2.273431264
GNB2	1.03414575	8.9907918	4.969606581	3.92E-05	0.02674804	2.213466448
HCK	1.2364042	9.70474544	4.962632941	3.99E-05	0.02674804	2.197205268
GBA	0.8926077	7.75524764	4.960280065	4.02E-05	0.02674804	2.191718332
SOCS3	1.4801694	7.14857808	4.889358043	4.83E-05	0.029113442	2.026220405
NFYC	0.6175825	7.7002732	4.88124924	4.94E-05	0.029113442	2.007286096
ARPC4	0.64320745	6.89827684	4.879187049	4.96E-05	0.029113442	2.002470443
ZFP36	0.70324215	11.99764908	4.877559351	4.98E-05	0.029113442	1.998669319
RTKN	0.8796891	1.61827712	4.867220128	5.12E-05	0.029113442	1.974522257
LIMK2	1.0285751	9.25329272	4.864320502	5.16E-05	0.029113442	1.967749584
EPB41L4A-AS1	-0.856914	10.611313	-4.838676436	5.51E-05	0.029329559	1.907840733
CXCR1	1.1258741	11.18019712	4.830320591	5.64E-05	0.029329559	1.888315663
BEST1	1.03362435	6.83233872	4.829397017	5.65E-05	0.029329559	1.886157424
XPO6	1.19489725	7.5656466	4.827316479	5.68E-05	0.029329559	1.881295461
PLEKHO2	0.801805	10.551081	4.808221377	5.97E-05	0.029329559	1.836666775
CEBPB	0.72393535	12.57134172	4.805715714	6.01E-05	0.029329559	1.830809845
BAZ2A	1.2252944	6.93015188	4.802794407	6.05E-05	0.029329559	1.823981143
SCARNA6	-1.34587825	4.9938804	-4.794808689	6.18E-05	0.029329559	1.805312989
IFNGR2	0.92295	8.5956594	4.792884701	6.21E-05	0.029329559	1.80081505
THOC5	1.07027815	6.41272448	4.784422885	6.35E-05	0.02944796	1.781031754
HIST1H3C	0.60061935	11.61051892	4.76241182	6.73E-05	0.029972002	1.729563048
TESK2	0.5940148	5.64317976	4.757854163	6.81E-05	0.029972002	1.718904479
CANT1	1.07398015	5.46427248	4.740764085	7.12E-05	0.029972002	1.678933682

SEC14L1P1	0.85546	7.641357	4.728059555	7.36E-05	0.029972002	1.649216288
CCDC13	1.33867765	2.60377328	4.723390668	7.45E-05	0.029972002	1.638294487
SNORD107	-0.97700435	4.51872628	-4.714026084	7.63E-05	0.029972002	1.616387058
NUDT16	1.16080675	4.9707126	4.701521556	7.88E-05	0.029972002	1.587131912
CHPF2	0.6127894	6.91181468	4.700775332	7.90E-05	0.029972002	1.585385999
CEP41	-0.66663935	5.13040468	-4.700313666	7.91E-05	0.029972002	1.584305853
BCAM	0.79549685	1.56051412	4.685224168	8.23E-05	0.029972002	1.548999787
CC2D2B	1.1151314	2.12537788	4.680159939	8.33E-05	0.029972002	1.537149966
GCA	0.74669455	13.75736236	4.676732934	8.41E-05	0.029972002	1.529130923
RPS29	-1.0813003	13.00063284	-4.671956272	8.51E-05	0.029972002	1.517953517
C10orf54	1.04852765	12.32663348	4.671294089	8.53E-05	0.029972002	1.516403988
RORB	-1.3178838	5.01307284	-4.669252266	8.57E-05	0.029972002	1.511626027
SLC16A3	0.8790101	13.32368812	4.661356209	8.75E-05	0.029972002	1.493148481
TMEM140	1.210117	8.0415226	4.648089732	9.06E-05	0.029972002	1.46210233
NUTM2F	0.7552736	3.97532312	4.645954296	9.11E-05	0.029972002	1.457104856
C5AR2	0.8898524	5.55967028	4.645950965	9.11E-05	0.029972002	1.457097062
MYL2	0.65016875	1.3022808	4.63924091	9.27E-05	0.029972002	1.441393576
TNIP2	0.85002655	5.62421256	4.636791018	9.33E-05	0.029972002	1.435660036
H3F3C	0.92216795	13.09861244	4.634457994	9.39E-05	0.029972002	1.430199972
CEACAM3	0.9707246	6.96983392	4.631512134	9.46E-05	0.029972002	1.423305616
APOBR	0.9275036	10.33583452	4.631310499	9.47E-05	0.029972002	1.422833715
NOTCH1	0.76459975	7.2933294	4.618965331	9.78E-05	0.03027266	1.393941136
TREML2	1.32344745	7.49854244	4.606585371	0.000100958	0.03027266	1.364966498
TNFRSF10C	0.9098626	13.14712492	4.602941632	0.000101921	0.03027266	1.356438435
RPS14	-0.7075465	10.9247288	-4.601210204	0.000102382	0.03027266	1.352386069
FNDC3B	0.6763501	7.65452692	4.598007249	0.00010324	0.03027266	1.344889616
HAUS4	0.96238595	6.59430744	4.59274209	0.000104666	0.03027266	1.332566586
AOAH-IT1	1.32172675	2.5000376	4.591790627	0.000104926	0.03027266	1.330339696
C19orf71	1.3762016	1.89147272	4.58883021	0.000105739	0.03027266	1.32341087
KREMEN1	1.64348865	4.49467668	4.582334863	0.000107543	0.03027266	1.308208572
TCEAL1	-0.703313	6.4319078	-4.581149845	0.000107876	0.03027266	1.305435053
DGAT2	1.45107245	8.26175824	4.576032393	0.000109324	0.03027266	1.293457727
PPP1R15A	1.2645481	6.59937412	4.575067923	0.000109599	0.03027266	1.291200401
SNORA57	-0.67845015	8.53892752	-4.564213368	0.000112743	0.030805134	1.265795633
ST3GAL4	0.76651	3.6611266	4.560295168	0.0001139	0.030805134	1.25662528
HSPA6	1.05494955	8.79426516	4.555135115	0.000115441	0.030900188	1.24454851
THEMIS2	1.12065005	9.48523996	4.550542252	0.000116831	0.030921341	1.233799305
LTBR	1.2302609	7.33372128	4.54703867	0.000117902	0.030921341	1.225599538
IRF1	0.85381615	10.02773868	4.540299452	0.00011999	0.031154319	1.209827286
LILRA2	1.100057	8.049919	4.531064541	0.000122912	0.031468178	1.188214654
FUT7	1.1871268	6.20449696	4.524681636	0.000124973	0.031468178	1.173276986
CDK5RAP3	0.8289064	5.25867808	4.521872311	0.000125891	0.031468178	1.166702534
TTN-AS1	0.76009315	5.38523448	4.518661951	0.000126948	0.031468178	1.159189654
NSFL1C	0.6913967	6.86643344	4.517721306	0.000127259	0.031468178	1.156988374

AFF1	0.89027595	6.25152724	4.509980798	0.000129851	0.031468774	1.138874506
WWC3	0.796766	8.7948118	4.508256408	0.000130435	0.031468774	1.134839274
RPL39	-1.26213475	12.6205784	-4.506898328	0.000130898	0.031468774	1.131661263
PHC2	1.1461883	10.04693976	4.500467835	0.000133108	0.031706671	1.116613691
DHX34	1.0863116	5.75438112	4.48787428	0.000137546	0.032174418	1.087145753
RAB5C	0.98822155	6.89257276	4.48786285	0.00013755	0.032174418	1.087119007
ATP8A2	-0.678102	2.1940696	-4.468777479	0.000144559	0.032265665	1.042464813
NFAM1	0.8189155	11.8750824	4.455373531	0.000149693	0.032265665	1.011106879
TMLHE	0.70915475	4.8729188	4.452587679	0.000150783	0.032265665	1.004589891
ATP6V1E1	1.1153786	6.68343912	4.447941586	0.000152617	0.032265665	0.993721525
HIST1H4B	-1.04924265	10.30464632	-4.447346605	0.000152854	0.032265665	0.992329747
SLC15A4	0.6625583	8.87382936	4.440296735	0.000155685	0.032265665	0.975839209
FLOT1	1.0461324	9.54307088	4.439836526	0.000155872	0.032265665	0.974762757
NUCB1	0.88398415	6.57126908	4.434983027	0.000157853	0.032265665	0.963410424
SERPINA1	0.9802905	7.6701122	4.430967951	0.000159512	0.032265665	0.954019524
RPS15A	-1.18366155	13.35134724	-4.425904896	0.000161627	0.032265665	0.942177987
CFAP58	1.276987	3.510273	4.425798743	0.000161672	0.032265665	0.941929721
ELF2	0.59103505	6.90493836	4.418199018	0.000164902	0.032265665	0.924156462
THNSL2	1.12135455	2.37580836	4.417350882	0.000165266	0.032265665	0.922173033
HCG27	0.84425385	7.77337852	4.416105273	0.000165803	0.032265665	0.919260112
NAP1L1	-0.76947815	10.68430472	-4.414348726	0.000166562	0.032265665	0.915152397
SLC12A9	0.7925318	9.10010256	4.414221069	0.000166617	0.032265665	0.914853872
LSM5	-1.0574482	8.46072176	-4.413366548	0.000166988	0.032265665	0.91285559
SLC9A8	1.02765145	7.62299004	4.409987004	0.000168463	0.032265665	0.904952752
SHOX2	1.5178884	2.37534868	4.409335901	0.000168749	0.032265665	0.903430223
SLC39A1	1.0363734	6.17923628	4.406832154	0.000169852	0.032265665	0.897575595
LTB4R	1.31107295	5.62364604	4.40476805	0.000170767	0.032265665	0.892749122
OR4K14	0.6710837	1.34688204	4.40401975	0.0001711	0.032265665	0.890999406
TUBA4A	0.81153055	10.32873956	4.403916065	0.000171146	0.032265665	0.890756965
MIDN	0.63107545	11.36948504	4.40278211	0.000171651	0.032265665	0.888105517
GPR39	1.20201345	2.20324924	4.401470775	0.000172238	0.032265665	0.885039356
SNHG6	-1.0542159	10.31812472	-4.400360631	0.000172736	0.032265665	0.882443654
NDUFA4	-1.14250605	11.79227744	-4.38789645	0.00017843	0.032802213	0.853302508
G6PD	1.7597324	6.47572968	4.387837297	0.000178457	0.032802213	0.853164219
BRI3	0.7652539	9.81137908	4.385813859	0.000179399	0.032802213	0.848433835
BAGE	0.721418	4.0687932	4.379073021	0.000182572	0.033149024	0.832675955
DYSF	1.2688929	8.16486128	4.370014236	0.000186925	0.033611167	0.811501525
NUAK2	0.7550986	11.29256292	4.366121778	0.000188827	0.033611167	0.802403858
LRP10	1.30122795	7.54877284	4.365767453	0.000189001	0.033611167	0.801575733
SNORD3B-1	-1.09641355	10.19781084	-4.347567947	0.000198161	0.034713941	0.759045311
COX7C	-1.35099805	12.18200164	-4.344140932	0.000199935	0.034713941	0.751037925
RAB24	0.81489025	11.0796448	4.342957897	0.00020055	0.034713941	0.748273796
SOD2	0.76851405	12.90446316	4.335065696	0.000204708	0.035198882	0.729835117
TNFRSF17	-1.70894865	5.98080412	-4.330347767	0.000207234	0.035398795	0.71881358

CKS2	-1.21767035	7.35894288	-4.313216207	0.000216669	0.036701765	0.678799409
RNF146	0.99596035	6.76447792	4.305165947	0.00022125	0.036900021	0.660000258
PLAUR	1.27056185	7.44533312	4.304370936	0.000221707	0.036900021	0.65814387
RHOXF2	0.81798395	1.45920764	4.287381357	0.000231712	0.038077015	0.61847847
MKRN9P	0.58701895	1.49182024	4.282844089	0.000234459	0.038286102	0.607887388
OSM	1.194389	4.7064704	4.278186675	0.000237312	0.038509838	0.597016774
SEMA4A	0.88690705	6.99465016	4.270698565	0.000241972	0.039022151	0.579541178
SIGLEC7	0.8628445	6.8065282	4.259252676	0.000249271	0.039835569	0.552833873
PRMT5	1.05188095	5.65612344	4.255642099	0.000251619	0.039835569	0.544410376
MICAL1	0.72781405	7.71464596	4.251395649	0.000254408	0.039850588	0.534504166
CSF2RB	0.9840205	6.1191904	4.250827511	0.000254783	0.039850588	0.533178868
ATP6V0E1	0.69937345	10.78314984	4.247578387	0.000256941	0.039911708	0.525599912
SLC22A12	0.7901419	1.81983848	4.245623177	0.000258249	0.039911708	0.521039406
CSNK1D	0.70622355	8.09494336	4.239339208	0.000262495	0.040327931	0.506383379
PEAR1	1.223572	2.1044612	4.236987739	0.000264102	0.040336107	0.500899579
IL10RB	0.87502125	8.9393726	4.233115962	0.000266769	0.040361066	0.491870909
TPD52L2	0.71765755	6.09131216	4.232242179	0.000267374	0.040361066	0.489833424
LPAR2	1.21895715	7.71661408	4.226471729	0.000271408	0.040733102	0.476378871
FLOT2	1.35374235	7.55376372	4.220995968	0.000275291	0.04074482	0.463613014
NABP1	0.7060009	9.84493588	4.218780587	0.000276878	0.04074482	0.458448654
FAM175A	-0.6118607	6.59198896	-4.217033376	0.000278135	0.04074482	0.454375848
HLX	1.381819	5.5240062	4.212113394	0.000281708	0.04074482	0.44290808
ZFAND3	0.5948867	6.23287084	4.212020914	0.000281775	0.04074482	0.442692535
RPL13AP3	-0.68020635	13.05725528	-4.210797297	0.000282671	0.04074482	0.439840669
RBX1	-1.01284855	10.94050624	-4.208934427	0.00028404	0.04074482	0.435499059
MRPL30	-0.5901229	8.78599932	-4.206180383	0.000286076	0.040811411	0.429080825
PITPNA-AS1	-0.6679964	7.88234912	-4.196197755	0.000293578	0.041441121	0.405820058
SELPLG	1.2474839	9.00023868	4.196061058	0.000293682	0.041441121	0.405501575
RAB4B	0.7390273	7.59447576	4.193377559	0.000295733	0.041504893	0.399249663
FFAR2	1.19778545	9.32493764	4.188051593	0.000299845	0.041597453	0.386842647
ZC3H3	0.6292319	9.62222848	4.185126636	0.000302127	0.041597453	0.380029557
SRMS	0.7447029	8.39130748	4.184376105	0.000302715	0.041597453	0.378281429
HINT1	-1.08718425	13.044537	-4.184266942	0.000302801	0.041597453	0.37802717
OS9	0.8617884	10.51407568	4.17685251	0.000308676	0.041891466	0.360759383
MVP	0.81962625	13.3983438	4.175846353	0.000309482	0.041891466	0.358416351
DBNL	0.90117435	8.35137212	4.175473638	0.000309781	0.041891466	0.357548429
AOAH	0.7163016	9.06080152	4.172771805	0.000311958	0.041967241	0.351257047
HIST2H2BF	1.21521965	4.69518288	4.169810103	0.000314361	0.042072585	0.344361051
NME1	-0.7440613	9.53007384	-4.166273727	0.000317255	0.042076104	0.336127687
MDFIC	-0.702067	6.8571868	-4.163394597	0.000319631	0.042076104	0.329425085
ABCA1	0.8626565	6.7317074	4.161480031	0.00032122	0.042076104	0.324968266
NDUFB2	-0.873967	11.8115202	-4.159956708	0.000322491	0.042076104	0.321422362
TMBIM1	0.93715245	8.55425844	4.152110641	0.000329112	0.042660359	0.303161049
ACTN4	0.6053018	8.54705956	4.14932021	0.0003315	0.042660359	0.296667405

ALPL	1.77659685	6.80891432	4.14885671	0.000331898	0.042660359	0.295588837
NUCKS1	-0.76310915	5.08246092	-4.142728351	0.000337207	0.043106696	0.281329401
ERF	0.95290415	5.37800628	4.141033403	0.000338691	0.043106696	0.277386035
CSF2RA	0.70988055	7.44198776	4.13757571	0.000341737	0.04328221	0.26934215
GADD45G	0.94336095	4.61728264	4.128658913	0.000349718	0.043591438	0.248602012
ARMC1	-0.6459814	7.65450172	-4.128421996	0.000349932	0.043591438	0.248051024
LOC100287036	0.8466619	1.87918748	4.125139537	0.000352918	0.043591438	0.240417542
TSEN34	0.6217579	12.72789908	4.120922124	0.000356792	0.043591438	0.230610867
UQCR11	-0.7687894	12.92555192	-4.120661872	0.000357032	0.043591438	0.230005749
UPP1	0.70096995	7.37991724	4.119247695	0.000358341	0.043591438	0.226717687
LILRA5	0.92522	7.7148478	4.112995413	0.000364186	0.043826599	0.212182344
LINC01031	0.7398686	1.59690192	4.11009835	0.000366927	0.043826599	0.205448161
OAZ2	0.6466361	8.37368552	4.107788621	0.000369126	0.043826599	0.200079651
GK3P	0.95004255	7.17445896	4.107665727	0.000369244	0.043826599	0.199794018
NDEL1	0.73294215	8.72438048	4.103928921	0.000372831	0.043826599	0.191109385
LOC400590	-0.89594225	10.1666726	-4.101736827	0.000374951	0.043826599	0.186015247
RPL24	-0.7950585	13.150627	-4.101085326	0.000375583	0.043826599	0.184501312
PFKFB3	1.27151945	6.15207964	4.09935632	0.000377267	0.043826599	0.180483656
VPS8	0.8221024	6.70226948	4.099051667	0.000377564	0.043826599	0.179775761
RBM47	0.84381485	7.49147272	4.098496787	0.000378107	0.043826599	0.178486457
OCIAD2	-0.6442389	9.85501432	-4.089822788	0.000386685	0.044621701	0.158334685
RPS17	-0.665152	7.9406128	-4.080812908	0.000395799	0.045271034	0.137408469
WDFY3	0.845253	8.1718316	4.078547562	0.000398124	0.04533722	0.132147968
MYD88	0.74898025	10.2481656	4.069102924	0.000407963	0.046254782	0.110220202
BST1	0.84831925	8.7647234	4.06525452	0.00041204	0.046513992	0.101287272
SIGLEC9	0.7755499	7.46980588	4.062447921	0.00041504	0.046649737	0.094773313
TMEM126A	-0.92440565	8.45200612	-4.046720679	0.00043225	0.048374711	0.05828282
RPL17	-1.2777389	12.13402672	-4.039146671	0.000440788	0.049118505	0.040716664
APOBEC3B	1.3205886	9.25268092	4.036441132	0.000443878	0.049215902	0.034442936
ZNF687	1.1735821	6.08838912	4.035069252	0.000445453	0.049215902	0.031261992
ORAI2	0.6208494	7.76920828	4.029420652	0.000451996	0.049506207	0.018166372
FMNL1	0.84313355	8.27859316	4.024260947	0.000458056	0.049506207	0.006206545
BOLA3	-0.87127015	6.66066612	-4.021941812	0.000460806	0.049506207	0.000831693

Supplementary Table 5: Immune score for each sample.

id	Immune score
GSM3526790_con	-5.527123053
GSM3526791_con	-4.661838585
GSM3526792_con	-1.739537327
GSM3526793_con	0.437852212
GSM3526794_con	1.001264316

GSM3526795_con	-0.159258906
GSM3526796_con	-3.424291045
GSM3526797_con	-2.01434156
GSM4548691_con	-1.563553451
GSM4548692_con	1.742410495
GSM4548693_con	-2.793214107
GSM4548694_con	-0.701259583
GSM4548695_con	-1.16953426
GSM4548696_con	-1.641291523
GSM4548697_con	-0.623811779
GSM4548698_con	-2.939887181
GSM4548699_con	-0.360181387
GSM4548700_con	-5.106663937
GSM4548701_con	0.37533865
GSM4548702_con	-2.329093916
GSM4548703_con	0.128241624
GSM4548704_con	0.432906286
GSM4548705_con	-0.970454905
GSM4548706_con	-0.608839767
GSM4548707_con	0.558112703
GSM3526782_treat	1.473518265
GSM3526783_treat	-0.557911754
GSM3526784_treat	2.467983668
GSM3526785_treat	-1.529709478
GSM3526786_treat	7.785799885
GSM3526787_treat	0.118037705
GSM3526788_treat	0.787463487
GSM3526789_treat	0.178238959
GSM4548708_treat	-2.817722574
GSM4548709_treat	-0.089403121
GSM4548710_treat	0.395322303
GSM4548711_treat	-0.087194403
GSM4548712_treat	-0.473353508
GSM4548713_treat	2.32736276
GSM4548714_treat	0.808065357
GSM4548715_treat	4.096580801
GSM4548716_treat	0.571857712
GSM4548717_treat	-2.411971808
GSM4548718_treat	-0.659490761
GSM4548719_treat	-0.768493875
GSM4548720_treat	5.089001373
GSM4548721_treat	2.482962402
GSM4548722_treat	5.182577381
GSM4548723_treat	6.332435839

GSM4548724_treat	2.956093371
